# The obesity pandemic and its impact on non-communicable disease burden

**DOI:** 10.1007/s00424-025-03066-8

**Published:** 2025-02-10

**Authors:** Staffan Hildebrand, Alexander Pfeifer

**Affiliations:** 1 Institute of Pharmacology and Toxicology, University Hospital, University of Bonn, 53127 Bonn, Germany; 2 PharmaCenter Bonn, University of Bonn, Bonn, Germany

**Keywords:** Obesity, Overweight, Non-communicable disease, Metaflammation

## Abstract

The rising prevalence of overweight and obesity across the globe is a major threat both to public health and economic development. This is mainly due to the link of obesity with the development and outcomes of non-communicable diseases (NCDs). NCDs are a leading cause of global death and disability, and reducing the burden of NCDs on patients and healthcare systems is of critical importance to improve public health. Obesity is projected to be the number one preventable risk factor for NCDs by 2035, and there is an urgent need to tackle the growing obesity rates in order to reduce NCD incidence and severity. Here, we review the current understanding of the impact of obesity on NCD burden in general, as well as the epidemiological and mechanistic relationship between obesity and some of the most common classes of NCDs. By literature review, we found that over 70% of NCDs have a documented association with obesity, highlighting the importance of a better understanding of the pathophysiologies underlying obesity/overweight as well as the interaction between obesity and NCDs in order to reduce global disease burden.

**Supplementary Information:**

The online version contains supplementary material available at 10.1007/s00424-025-03066-8.

## Introduction

Non-communicable diseases (NCDs) remain the largest contributors to excess death globally. According the World Health Organization (WHO), NCDs are responsible for 41 million deaths per year—a staggering 74% of all global deaths [117]. In terms of mortality, the six main classes of NCDs are cardiovascular disease (CVD), cancer, chronic respiratory diseases (CRD), diabetes and kidney diseases, digestive diseases, and neurological disease. In addition to being leading causes of excess death, NCDs are major contributors to global disease burden, with an estimated 1.62 billion disability-adjusted life years (DALYs) attributed to NCDs in 2019 [34]. This includes not only the six high-mortality classes of NCDs, but also other disease classes that lead to a significant reduction in quality of life, e.g., mental and musculoskeletal disorders. To date, there is no definite cure for the vast majority of NCDs, and long-term treatment is often required for disease management. The high incidence combined with a high cumulative treatment cost results in a tremendous financial burden on health systems and economic development worldwide, with an estimated total cost of $30 trillion globally between 2011 and 2030.

The causes of NCDs vary, but aside from genetic disorders, they are generally a combination of genetic predisposition and lifestyle factors. Strikingly, almost half (48%) of global deaths can be attributed to risk factors associated with an obesogenic lifestyle or predisposition (dietary and metabolic risk factors as well as low physical activity) [34]. Simply being overweight (defined by the WHO as a BMI ≥ 25) incurs a significant risk of death and disease by itself: high (BMI ≥ 25) body mass index (BMI) is associated with 8.52% of all deaths globally [16], and the WHO estimates that overweight and obesity are the fourth most common risk factors for NCDs in Europe [18].

Overweight and obesity (defined by the WHO as BMI ≥ 30) result from an imbalance in energy intake and expenditure. Excess nutrients are stored in adipose tissues—a physiological function which serves as a crucial buffer to ensure energy availability in times of need (e.g., famine). However, a chronic net caloric surplus will lead to uncontrolled adipose tissue hypertrophy beyond the limit of healthy adipose tissue expansion. This leads to adipocyte stress and consequently to adipose tissue inflammation and fibrosis. If not controlled, this local inflammation will spill over to other organs and will affect the whole body (a process also known as “Metaflammation”) with detrimental systemic consequences, such as insulin resistance and hyperglycemia, high blood triglycerides, low LDL levels, and hypertension. Each of these conditions may constitute a disease on their own, but often several occur simultaneously. A coincidence of at least three of these conditions is collectively termed metabolic syndrome.

**Fig. 1:**
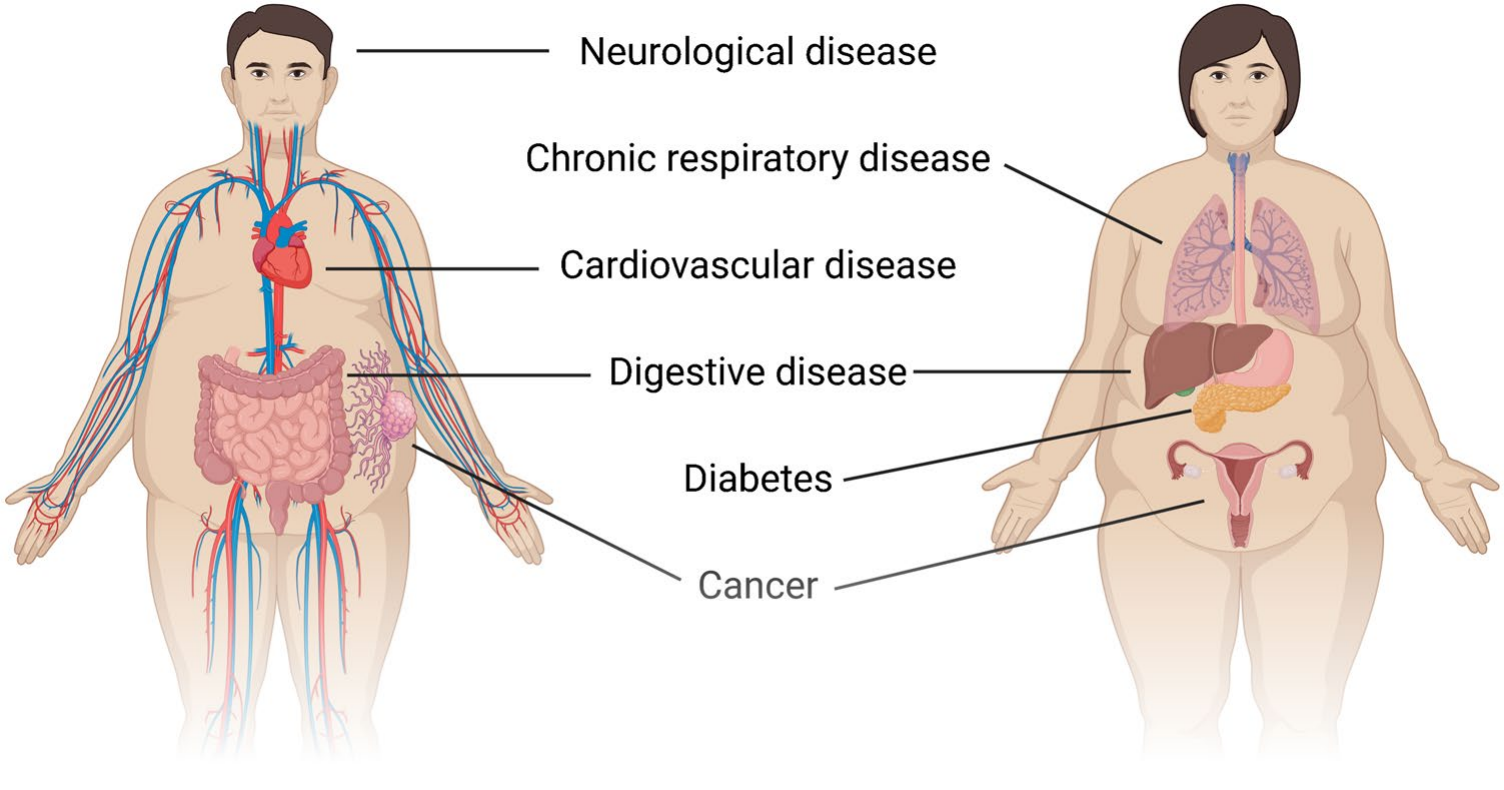
Scheme of different classes of NCDs which have significant association with overweight and obesity. Image created with BioRender (https://www.biorender.com/)

Over the last decades, overwhelming evidence has been published linking obesity with a plethora of disorders and diseases. Although for some diseases obesity appears to be inconsequential or even protective, the pathogenesis of the vast majority of NCDs is negatively affected by excess adiposity in some way. A literature review of each NCD included in the Global Burden of Disease [34] 2019 study showed a positive association with obesity for 71 out of 95 disorders/diseases (74.7%), while only six showed a negative association (6.3%) (Fig. 1, Supplementary Table 1).

Overweight and obesity already affect a significant portion of the global population with 43% being overweight and 16% being obese [118]. High-income countries are particularly affected: the corresponding rates for adults in the USA are 73.6% and 42.5% [27], while the rates in Europe are 59% overweight and 23% obese [18]. Alarmingly, the increasing obesity rates are evident also in low- and middle-income countries, which have fewer resources at hand to cope with the concomitant load on healthcare services. Thus, preventing or mitigating the increasing obesity rates would be an important strategy for reducing the severe burden of NCDs on global health and healthcare systems.

Here, we summarize the broad effects of obesity and metabolic syndrome on the incidence and progression of the most common classes of NCDs.

## Cardiovascular diseases

Cardiovascular diseases are the number one cause of death globally, accounting for almost a third of all deaths and almost 400 million DALYs [34]. Because of its grave consequences, high incidence, and strong correlation with obesity, cardiovascular disease is often considered the most important NCD associated with obesity.

### Ischemic heart disease and stroke

Several large-scale epidemiological studies have established both a correlation as well as a causal link between obesity and ischemic heart disease and stroke. The prospective Nurses’ Health Study identified obesity a significant independent risk factor for coronary disease in middle-aged women from the USA [73], and further meta-analysis has demonstrated similar results in large cohorts of men and women in other regions of the world [9, 81]. Recently, Censin et al. [15] performed Mendelian randomization analysis of a cohort of more than 400,000 men and women, revealing a direct causal relationship between obesity and both coronary heart disease and ischemic stroke.

Obesity and atherosclerosis are intimately connected by sharing a major root cause: overnutrition. Chronic overnutrition leads to increased concentrations of very low-density lipoprotein (VLDL) and chylomicrons in the blood over time and consequently to a higher concentration of atherogenic and cholesterol-rich intermediate density lipoprotein (IDL), low density lipoprotein (LDL), and chylomicron remnants. The deposition of LDL- and chylomicron-derived cholesterol in sub-endothelial space of the vascular wall, followed by inflammation of the surrounding tissue and formation of atherosclerotic lesions, is a key initiating step in the formation of atherosclerotic plaques [10, 58]. Simultaneously, the increased levels of triglyceride-rich lipoproteins in the blood enhances the activity of cholesteryl ester transfer protein (CETP), leading to blunted reverse cholesterol transport by increasing the exchange of cholesterol for triglycerides from HDL to LDL and VLDL, as well as lower HDL levels [58]. Additionally, chronically elevated serum nutrients (especially glucose and saturated fat) can trigger endothelial dysfunction and reduce insulin sensitivity in metabolically relevant organs, leading to impaired capacity for lipoprotein and glucose clearance, further enhancing atherogenesis [55, 58, 99, 114].

Furthermore, obesity (particularly central obesity) often leads to a state of systemic inflammation (metaflammation, see above) further enhanced by spill-over of lipids to non-adipose organs like the liver [119]. The increased levels of circulating inflammatory cytokines may also worsen the progression and stabilization of atherosclerotic plaques [44, 100, 102].

### Hypertensive heart disease

The association between adiposity and hypertension has been recognized since the 1960s [51]. Hypertension increases the force required for the heart to expel blood, and chronic pressure overload can lead to ventricular thickening, hypoxia, and heart failure (hypertensive heart disease). Obesity is a major cause of hypertension, with an estimated 65–78% of hypertension cases attributed to overweight or obesity [108]. Generally, obesity is thought to initiate hypertension by impairing kidney function, via mechanisms including physical stress (compression) of the kidneys by abdominal adipose tissue, sympathetic nervous system (SNS) activation, and activation of the renin–angiotensin–aldosterone system [40]. Obesity often also leads to masked hypertension and increased pulse pressure due to increased cardiac output during everyday activities, which is not sufficiently compensated by arterial compliance [20].

### Aortic stenosis

Aortic stenosis, the gradual calcification and thickening of the aortic valves, is a common vascular disease in elderly subjects affecting more than a tenth of the population above the age of 75 [87]. Aortic stenosis is a progressive disease which ultimately leads to death unless treated. Currently, the only therapeutic option for patients with aortic stenosis is aortic valve replacement. Although the precise etiology of non-rheumatic aortic valve calcification and stenosis is not fully understood, obesity has been demonstrated to constitute a significant risk factor for aortic stenosis development [60].

Recently, a direct causal link between obesity and aortic stenosis could also be established. By Mendelian randomization analysis of a cohort of over 100,000 individuals, Kaltoft et al. [50] demonstrated that an increase in BMI of only 1 kg/m^2^ increases the risk of aortic stenosis by over 50% (causal risk ratio 1.52).

### Aortic aneurysm

Like aortic stenosis, the initiating causes of aortic aneurysms are poorly understood. The majority of aortic aneurysms occur in the abdominal aorta, affecting 4.6% of men and 1.2% of women over the age of 45 [74]. Untreated aortic aneurysm can lead to dissection and rupture of the aorta. Obesity has been associated with an increased incidence of abdominal aortic aneurysm [36], as well as increased risk of dissection in elderly patients [110].

Aneurysm of the thoracic aorta is less common but is a relatively frequent complication in patients with Marfan’s disease. A recent study surprisingly demonstrated that obesity is frequent in Marfan’s disease patients, and that obese patients with Marfan’s disease more often suffer from aortic complications [121], although a causal relationship has not yet been established.

## Chronic respiratory diseases

Obesity has been shown to increase the risk of childhood asthma [31, 35, 122], and weight loss intervention has been shown to improve lung function and asthma control in asthmatic children as well as adults [93]. Surprisingly, maternal obesity also significantly increases the risk of developing asthma by 2–3% for each maternal BMI point [24]. The precise mechanism behind the link between obesity and asthma is not known. However, adipose tissue inflammation is increased in obese asthmatic patients compared to obese controls [111], and circulating IL-6, which is in part produced by adipose tissue and is elevated in obesity [21, 76], is associated with asthma severity [92]. Additionally, metabolic dysfunction is a stronger predictor of asthma development than fat mass in obesity [93]. These findings suggest an important role of inflammation in obesity-related asthma.

## Diabetes

Type II diabetes (T2D), or adult-onset diabetes, is the most common comorbidity in obesity: severe obesity incurs a lifetime risk of developing T2D of 70–75% [80]. Furthermore, 80% of patients with T2D are obese [8]. The T2D-related burden on individuals as well as health care systems is enormous: more than 6% of the world’s population have T2D, and over 1 million deaths per year can be attributed to the disease [53]. As with atherosclerosis, the causal link between obesity and T2D is well understood. The precise mechanisms behind T2D development in obesity are less clear, but evidence suggests a combination of at least three factors: (1) increased insulin resistance due to chronic adipose tissue inflammation and dysregulation of adipokine secretion (e.g., adiponectin and plasminogen activator-1); (2) increased hepatic gluconeogenesis; and (3) pancreatic β-cell dysfunction [57].

A link between obesity like T2D appears to be logical; however, there is also a connection between obesity and Type I diabetes. Magnus et al. [71] showed that both paternal and maternal obesity increases the likelihood of T1D development in the offspring. Interestingly, this was not linked to maternal gestational body weight, suggesting that parental lifestyle may be a confounding factor. Additionally, Zucker et al. [123] found that a BMI increase of 5 kg/m^2^ during adolescence increases the risk of T1D by 35% in early adulthood. How adiposity may increase the risk of autoimmunity is still unknown.

## Digestive diseases

### Hepatosteatosis and cirrhosis

Metabolic dysfunction-associated steatotic liver disease (MASLD), formerly known as non-alcoholic fatty liver disease (NAFLD), is characterized by excessive fat accumulation in the liver. If left untreated, it can progress into metabolic dysfunction-associated steatohepatitis (MASH) and liver failure. This occurs through multiple pathways that induce lipotoxicity/lipid spillover and consequently inflammatory activation, e.g., ER and oxidative stress and mitochondrial dysfunction [68]. In turn, MASH can lead to further complications such as fibrosis, cirrhosis, and hepatocellular carcinoma. Overnutrition is the primary cause of MASLD, and obesity or overweight is one of the five cardiometabolic criteria for MASLD diagnosis. The prevalence of MASLD in overweight and obese patients is around 70–75% [97]. The degree and prevalence of steatosis increases with BMI, and for morbidly obese patients, the rate of steatosis has been reported to be above 90%. Cirrhosis, i.e., irreversible end-stage liver disease, has been reported in 9–12% of morbidly obese patients [2, 85]. As mentioned above (see *Ischemic heart disease and stroke*), caloric intake exceeding the physiological capacity of storage in adipose tissues leads to spill-over in other organs, including the liver. Consequently, weight loss leads to a reduction in hepatic steatosis in MASLD patients in a dose-dependent manner [22].

### Inflammatory bowel disease

The major types of inflammatory bowel disease are ulcerative colitis (UC) and Crohn’s disease (CD). Neither disease have a known cause, and while the symptoms (e.g., abdominal pain, diarrhea, fever, and weight loss) are similar, they are distinct diseases with differing etiology. CD is typically characterized as a chronic inflammatory disorder, while UC is believed to be an autoimmune disease. Data obtained from the Nurses Health Study II revealed a significant association with obesity and CD [52], and other studies have shown a more rapid clinical course in overweight patients compared to controls [41]. The impact of obesity on CD development seems to be more pronounced when obesity is established at a young age [43]. As the fundamental cause of CD is unknown, it is also not clear how obesity increases the risk of it. However, mechanisms such as gut inflammation via intestinal dysbiosis, and obesity-related systemic inflammation, have been proposed [56]. The same analysis of the Nurses Health Study II that revealed an association between obesity and CD could not identify any increased risk of UC in obese subjects [52].

### Pancreatitis

Gallstones are one of the most common risk factors for acute pancreatitis and is the primary cause of the disease in 38% of patients [115]. As obesity (especially central obesity) is a major risk factor for developing gallstones [90], obesity also increases the risk of acute pancreatitis [116]. However, other consequences of obesity are independent risk factors for acute pancreatitis. Hypertriglyceridemia, which is common in obese patients, can directly cause acute pancreatitis [30], while type 2 diabetes increases the risk of developing the disease [32]. Additionally, obesity has been shown to increase the severity of acute pancreatitis regardless of the cause [54].

## Neurological diseases

Overweight and obesity have been linked to cognitive impairment in numerous studies [112], as a consequence of brain atrophy, neuroinflammation, hypoperfusion, and altered brain metabolism [25, 83]. Alarmingly, there is also mounting evidence that adiposity and its co-morbidities influence both the development and progression of the most common neurodegenerative diseases.

### Alzheimer’s disease

Several studies have identified an association between mid-life obesity and Alzheimer’s disease (AD) diagnosis later in life [95]. In contrast, low BMI has been associated with an increased rate of AD diagnosis at a 1–3 year follow-up in elderly patients [82]. However, it has been suggested that this may be due to weight loss being an early consequence of disease onset. In a study with a shorter follow-up time (18 years), obesity at 70 and 79 years was associated with an increase in AD incidence in women [39].

Interestingly, diabetes also increases the risk of AD. A meta-analysis pooling the effects of obesity, diabetes, and abnormal glucose and insulin levels found stronger effect size for AD than obesity alone, suggesting that metabolic syndrome as a consequence of obesity might be a stronger risk factor for AD that obesity per se [96].

### Parkinson’s disease

Although several studies examining a potential relationship between BMI and the risk of Parkinson’s disease (PD) have not consistently found an association with obesity and PD [1, 69, 88, 101]. However, abdominal obesity (waist circumference) and body shape index have been found to significantly increase the risk of PD [47, 89]. Importantly, high waist circumference is associated with increased PD risk even in normal weight patients [89], suggesting that abdominal obesity, but not high BMI per se, is a risk factor for PD. Interestingly, type 2 diabetes, which is strongly correlated with abdominal obesity, is also a strong risk factor for PD [6].

### Multiple sclerosis

In contrast to PD, the connection between obesity and multiple sclerosis (MS) is clear. Obesity in either childhood, adolescence and early adulthood, has been shown to significantly increase the risk of MS later in life [42, 77, 78]. Importantly, a recent Mendelian randomization study found a causal association between both BMI and visceral adiposity [75]. Obesity seems to not only increase the risk of developing MS, but also to affect disease outcome, and there is an association between high BMI and disease severity as well as poorer outcomes [70].

### Amyotrophic lateral sclerosis

Amyotrophic lateral sclerosis (ALS) is one of the few examples of NCDs in which obesity appears to be protective. Several studies have demonstrated a positive correlation between ALS survival and obesity or an inverse correlation between BMI and ALS progression [19, 28, 67]. Surprisingly, even diagnosis of type 2 diabetes may be associated with a reduced ALS rate. The cause of this association is unknown, but since weight loss is common during ALS progression and is associated with poor prognosis [49, 67, 79], it is possible that a larger “energy reservoir” at disease onset could prolong the health span of ALS patients.

## Cancer

There is a well-established link between many types of cancer and obesity. With some exceptions (e.g., testicular cancer and non-melanoma skin cancer), obesity correlates positively with the incidence of most types of neoplasms (see Supplementary Table 1). Globally, 4–8% of all new cancer diagnoses in adults and between 5 and 20% of all cancer deaths have been attributed to obesity [5, 13, 34, 113]. However, some cancers show particularly strong association with increased BMI—especially gastrointestinal, uterine, kidney, pancreatic, and breast cancers [4, 5, 61]. It is not known exactly how obesity increases the risk of cancer, but several mechanisms have been proposed. In obesity, increased aromatase activity enhances estradiol production, which can have a pro-mitogenic effect in some tumors. Obesity also often leads to elevated levels of insulin and free IGF-1, which also enhances mitogenesis in cancer cells [107]. Additionally, altered secretion of pro- and anti-inflammatory cytokines and adipokines may lead to increased oxidative stress and DNA damage, resulting in higher likelihood of tumorigenesis [91]. Here, we are focusing on cancers of the GI tract, gynecological cancers, and breast cancer.

### Esophageal cancer

The two most common esophageal cancers are esophageal adenocarcinoma (EAC) and esophageal squamous cell carcinoma. Obesity is a major risk factor for EAC, with severely obese patients exhibiting an almost fivefold increased risk of EAC development [61]. This may at least partially be explained by increased rates of gastroesophageal reflux disease (GERD) and Barret’s esophagus. Moreover, one meta-study using pooled data from 12 epidemiological studies found significant correlation between obesity and EAC even in patients without diagnosed GERD [46].

### Colorectal cancer

Colorectal cancer (CRC) ranks third in cancer prevalence and second as a cause of cancer-associated deaths globally [33]. An increase in body weight of 10 kg increases the risk of developing CRC by 8%. Both weight gain in adulthood as well as early-life obesity significantly increases the risk of CRC [29, 106]. Interestingly, bariatric surgery for weight loss has been shown to reduce the risk by approximately 27% [120]. The exact mechanisms that link overweight/obesity with CRC are so far unknown, but it has been speculated to be due to systemic inflammation, dysregulated adipokine secretion, and altered gut microbiota [120]. Moreover, it has been suggested that the correlation between obesity and CRC may be underestimated, as many patients lose weight prior to cancer diagnosis, and the reported BMI in many studies is measured close to diagnosis [72].

### Gynecological cancers

Among the cancers affecting the female reproductive system, carcinoma of the endometrium is the most common and shows the strongest correlation with obesity [109]. Obesity increases both the risk of developing endometrial cancer, as well as the severity of the disease once diagnosed. For each 5 kg/m^2^ of BMI, the risk of developing endometrial cancer increases by roughly 50%, and the relative risk of mortality after diagnosis is 2.53 for obese women, and 6.25 for morbidly obese women. As mentioned above, adipose tissues express aromatase, and total aromatase activity is increased in obesity. Estrogens act as powerful mitogenic and mutagenic factors in endometrial tissue, and the increased levels of estrogens in obese postmenopausal women have been proposed as one of the causal mechanisms behind the strong correlation between obesity and endometrial cancer [86].

Other gynecological cancers have a less clear association with obesity: While some studies have identified obesity as a risk factor for ovarian cancer, the overall evidence is weak [23, 84]. Overweight and obesity have been associated with increased incidence of cervical cancer, but recent studies suggest that this might be due to underdiagnosis of cervical precancer in obese individuals, as well as lower rates of participation in cancer screening [17, 104].

### Breast cancer

The relationship between obesity and breast cancer appears to depend largely on cancer type and menopausal status. In premenopausal women, obesity is inversely correlated with hormone receptor-positive (HR+) breast cancer risk. In postmenopausal women, however, obesity is positively correlated with HR+ cancer risk [94]. Interestingly, obesity in premenopausal women is associated with a decreased level of circulating estradiol, while the opposite is true for postmenopausal women [26]. In this context, it is of interest that aromatase-derived estrogen from peripheral tissues (especially adipose tissue) is the major source of circulating estradiol after menopause [38]. This difference in adipose tissue-derived estrogen production provides an intriguing explanation for the contrasting risk ratios of HR+ breast cancer in pre-and post-menopausal women. In contrast, HR- breast cancer incidence is strongly correlated to obesity in premenopausal women, but not in postmenopausal women, while obesity increases the risk of inflammatory breast cancer in both patient groups [94]. Obesity is associated with poorer outcomes regardless of breast cancer types and patient groups [64]. Other obesity-related mechanisms, such as low-grade systemic inflammation and increased secretion of pro-angiogenic and pro-mitogenic cytokines, have been proposed to contribute to the over-all detrimental effects of obesity in breast cancer [94].

### Pancreatic cancer

Several meta-analyses have found a significantly increased risk of pancreatic cancer in obese individuals [7, 59, 98]. Pancreatic cancer is the 11th most common type of cancer and has an extremely poor prognosis with a survival rate of around 6% [48]. Interestingly, weight loss induced by bariatric surgery has been shown to significantly reduce the risk of pancreatic cancer [103].

## The obesity paradox

Although an obese state generally causes an increased metabolic, inflammatory, and mechanical load on the body, increased BMI can sometimes be beneficial. This effect is known as the obesity paradox. In some cases, the different effects of obesity can be easily explained on a purely mechanical basis: for example, obesity is strongly associated with increased lower back pain, but not neck pain, clearly reflecting the mass distribution in obese patients and the resulting mechanical load of the relevant body parts. Similarly, the positive correlation of obesity with abdominal hernias, but negative correlation with inguinal and femoral hernias, may also be explained in anatomical terms. In other cases, the differences are not easily understood. The abovementioned discrepancy between ALS and other neurological disorders has so far defied explanation, and the mechanism behind the protective effect of obesity on squamous cell carcinoma of the esophagus, but detrimental effect on esophageal adenocarcinoma is likewise unclear.

The obesity paradox has been recognized in CVD patients for over two decades yet remains controversial [62]. Originally described in heart failure patients, a beneficial effect of moderately increased BMI, body fat, or waist circumference has also been shown in patients with coronary artery disease and peripheral artery disease [63]. However, recent research using updated and improved adiposity indices (e.g., hip-to-waist ratio) found no protective effect of increased adiposity [12]. Possibly, the earlier reports on the obesity paradox in CVD patients may have been a consequence of reduced cardiovascular fitness, sarcopenia, and increased frailty (by having less stored energy reserves) in the patients in the non-obese groups [14, 65].

Similarly, the obesity paradox has also been observed among cancer patients. While it is clear that obesity is a risk factor for developing many types of cancers, several studies have shown a correlation between BMI and overall survival in cancer patients after diagnosis [11, 45, 105]. However, BMI may be a poor read-out for adiposity in this case: sarcopenic patients with obesity have been shown to have the worst prognosis among cancer patients, suggesting that there might be no cancer obesity paradox when using more appropriate measures for adiposity [37]. Additionally, the apparent cancer obesity paradox has been suggested to be a product of methodological issues, including selection bias, collider stratification bias, and reverse causality [66].

Interestingly, in some cases, maternal obesity may also protect the offspring from developing NCDs. Alam et al. [3] found that maternal obesity reduces the risk of testicular cancer in the offspring, through so far unknown mechanisms.

## Conclusions

There is a clear link between NCDs and obesity: Obesity is the leading cause for NCDs. Importantly, obesity is linked to those NCDs with grave consequences. The spectrum of diseases ranges from cardiovascular disease (myocardial infarction, stroke, hypertension, leading to sudden death and heart failure), metabolic disorders (as expected), and certain cancers (e.g., colorectal and gynecological cancers).

Thus, it is of outmost importance to tackle obesity, and there is an urgent need for pharmacological therapies. The new incretin-based drugs (“ozempic”) have enormous success reaching almost levels of bariatric surgery. However, these are considered to be “forever” drugs, and other novel approaches are urgently needed for alternative and synergistic treatments.

## Supplementary Information

Below is the link to the electronic supplementary material.

Supplementary file1 (DOCX 187 kb)

## Data Availability

No datasets were generated or analysed during the current study.

## References

[1] Abbott RD, Ross GW, White LR, Nelson JS, Masaki KH, Tanner CM, Curb JD, Blanchette PL, Popper JS, Petrovitch H (2002) Midlife adiposity and the future risk of Parkinson’s disease. Neurology 59(7):1051–1057. 10.1212/wnl.59.7.1051 12370461 10.1212/wnl.59.7.1051

[2] Abrams GA, Kunde SS, Lazenby AJ, Clements RH (2004) Portal fibrosis and hepatic steatosis in morbidly obese subjects: a spectrum of nonalcoholic fatty liver disease. Hepatology 40(2):475–483. 10.1002/hep.20323 15368453 10.1002/hep.20323

[3] Alam SS, Cantwell MM, Cardwell CR, Cook MB, Murray LJ (2010) Maternal body mass index and risk of testicular cancer in male offspring: a systematic review and meta-analysis. Cancer Epidemiol 34(5):509–515. 10.1016/j.canep.2010.07.006 20800565 10.1016/j.canep.2010.07.006 PMC3069655

[4] Aleman JO, Eusebi LH, Ricciardiello L, Patidar K, Sanyal AJ, Holt PR (2014) Mechanisms of obesity-induced gastrointestinal neoplasia. Gastroenterology 146(2):357–373. 10.1053/j.gastro.2013.11.051 24315827 10.1053/j.gastro.2013.11.051 PMC3978703

[5] Arnold M, Pandeya N, Byrnes G, Renehan PAG, Stevens GA, Ezzati PM, Ferlay J, Miranda JJ, Romieu I, Dikshit R, Forman D, Soerjomataram I (2015) Global burden of cancer attributable to high body-mass index in 2012: a population-based study. Lancet Oncol 16(1):36–46. 10.1016/S1470-2045(14)71123-4 25467404 10.1016/S1470-2045(14)71123-4 PMC4314462

[6] Athauda D, Evans J, Wernick A, Virdi G, Choi ML, Lawton M, Vijiaratnam N, Girges C, Ben-Shlomo Y, Ismail K, Morris H, Grosset D, Foltynie T, Gandhi S (2022) The impact of type 2 diabetes in Parkinson’s disease. Mov Disord 37(8):1612–1623. 10.1002/mds.29122 35699244 10.1002/mds.29122 PMC9543753

[7] Berrington de Gonzalez A, Sweetland S, Spencer E (2003) A meta-analysis of obesity and the risk of pancreatic cancer. Br J Cancer 89(3):519–523. 10.1038/sj.bjc.6601140 12888824 10.1038/sj.bjc.6601140 PMC2394383

[8] Bloomgarden ZT (2000) American Diabetes Association Annual Meeting, 1999: diabetes and obesity. Diabetes Care 23(1):118–124. 10.2337/diacare.23.1.118 10857981 10.2337/diacare.23.1.118

[9] Bogers RP, Bemelmans WJ, Hoogenveen RT, Boshuizen HC, Woodward M, Knekt P, van Dam RM, Hu FB, Visscher TL, Menotti A, Thorpe RJ Jr, Jamrozik K, Calling S, Strand BH, Shipley MJ, Investigators B-CC (2007) Association of overweight with increased risk of coronary heart disease partly independent of blood pressure and cholesterol levels: a meta-analysis of 21 cohort studies including more than 300 000 persons. Arch Intern Med 167(16):1720–1728. 10.1001/archinte.167.16.1720 17846390 10.1001/archinte.167.16.1720

[10] Boren J, Williams KJ (2016) The central role of arterial retention of cholesterol-rich apolipoprotein-B-containing lipoproteins in the pathogenesis of atherosclerosis: a triumph of simplicity. Curr Opin Lipidol 27(5):473–483. 10.1097/MOL.0000000000000330 27472409 10.1097/MOL.0000000000000330

[11] Brunner AM, Sadrzadeh H, Feng Y, Drapkin BJ, Ballen KK, Attar EC, Amrein PC, McAfee SL, Chen YB, Neuberg DS, Fathi AT (2013) Association between baseline body mass index and overall survival among patients over age 60 with acute myeloid leukemia. Am J Hematol 88(8):642–646. 10.1002/ajh.23462 23619915 10.1002/ajh.23462 PMC4214755

[12] Butt JH, Petrie MC, Jhund PS, Sattar N, Desai AS, Kober L, Rouleau JL, Swedberg K, Zile MR, Solomon SD, Packer M, McMurray JJV (2023) Anthropometric measures and adverse outcomes in heart failure with reduced ejection fraction: revisiting the obesity paradox. Eur Heart J 44(13):1136–1153. 10.1093/eurheartj/ehad083 36944496 10.1093/eurheartj/ehad083 PMC10111968

[13] Calle EE, Rodriguez C, Walker-Thurmond K, Thun MJ (2003) Overweight, obesity, and mortality from cancer in a prospectively studied cohort of U.S. adults. N Engl J Med 348(17):1625–38. 10.1056/NEJMoa021423 12711737 10.1056/NEJMoa021423

[14] Carbone S, Canada JM, Billingsley HE, Siddiqui MS, Elagizi A, Lavie CJ (2019) Obesity paradox in cardiovascular disease: where do we stand? Vasc Health Risk Manag 15:89–100. 10.2147/VHRM.S168946 31118651 10.2147/VHRM.S168946 PMC6503652

[15] Censin JC, Peters SAE, Bovijn J, Ferreira T, Pulit SL, Magi R, Mahajan A, Holmes MV, Lindgren CM (2019) Causal relationships between obesity and the leading causes of death in women and men. PLoS Genet 15(10):e1008405. 10.1371/journal.pgen.1008405 31647808 10.1371/journal.pgen.1008405 PMC6812754

[16] Chen Y, Ma L, Han Z, Xiong P (2024) The global burden of disease attributable to high body mass index in 204 countries and territories: findings from 1990 to 2019 and predictions to 2035. Diabetes Obes Metab 26(9):3998–4010. 10.1111/dom.15748 38957939 10.1111/dom.15748

[17] Clarke MA, Fetterman B, Cheung LC, Wentzensen N, Gage JC, Katki HA, Befano B, Demarco M, Schussler J, Kinney WK, Raine-Bennett TR, Lorey TS, Poitras NE, Castle PE, Schiffman M (2018) Epidemiologic evidence that excess body weight increases risk of cervical cancer by decreased detection of precancer. J Clin Oncol 36(12):1184–1191. 10.1200/JCO.2017.75.3442 29356609 10.1200/JCO.2017.75.3442 PMC5908221

[18] Copenhagen: WHO Regional Office for Europe (2022) WHO European Regional Obesity Report 2022. https://iris.who.int/bitstream/handle/10665/353747/9789289057738-eng.pdf Accessed 11 Dec 2024

[19] Dardiotis E, Siokas V, Sokratous M, Tsouris Z, Aloizou AM, Florou D, Dastamani M, Mentis AA, Brotis AG (2018) Body mass index and survival from amyotrophic lateral sclerosis: a meta-analysis. Neurol Clin Pract 8(5):437–444. 10.1212/CPJ.0000000000000521 30564498 10.1212/CPJ.0000000000000521 PMC6276330

[20] de Simone G, Mancusi C, Izzo R, Losi MA, Aldo Ferrara L (2016) Obesity and hypertensive heart disease: focus on body composition and sex differences. Diabetol Metab Syndr 8:79. 10.1186/s13098-016-0193-x 27956942 10.1186/s13098-016-0193-x PMC5129668

[21] El-Mikkawy DME, EL-Sadek MA, EL-Badawy MA, Samaha D (2020) Circulating level of interleukin-6 in relation to body mass indices and lipid profile in Egyptian adults with overweight and obesity. Egypt Rheumatol Rehabil 47(1):ARTN 7. 10.1186/s43166-020-00003-8

[22] Finer N (2022) Weight loss interventions and nonalcoholic fatty liver disease: optimizing liver outcomes. Diabetes Obes Metab 24(Suppl 2):44–54. 10.1111/dom.14569 34622555 10.1111/dom.14569

[23] Foong KW, Bolton H (2017) Obesity and ovarian cancer risk: a systematic review. Post Reprod Health 23(4):183–198. 10.1177/2053369117709225 28720017 10.1177/2053369117709225

[24] Forno E, Young OM, Kumar R, Simhan H, Celedon JC (2014) Maternal obesity in pregnancy, gestational weight gain, and risk of childhood asthma. Pediatrics 134(2):e535–e546. 10.1542/peds.2014-0439 25049351 10.1542/peds.2014-0439 PMC4187236

[25] Forny-Germano L, De Felice FG, Vieira M (2018) The role of leptin and adiponectin in obesity-associated cognitive decline and Alzheimer’s disease. Front Neurosci 12:1027. 10.3389/fnins.2018.01027 30692905 10.3389/fnins.2018.01027 PMC6340072

[26] Freeman EW, Sammel MD, Lin H, Gracia CR (2010) Obesity and reproductive hormone levels in the transition to menopause. Menopause 17(4):718–726. 10.1097/gme.0b013e3181cec85d 20216473 10.1097/gme.0b013e3181cec85d PMC2888623

[27] Fryar CDCM, Afful J (2020) Prevalence of overweight, obesity, and severe obesity among adults aged 20 and over: United States, 1960–1962 through 2017–2018. Accessed 11(12):2024

[28] Gallo V, Wark PA, Jenab M, Pearce N, Brayne C, Vermeulen R, Andersen PM, Hallmans G, Kyrozis A, Vanacore N, Vahdaninia M, Grote V, Kaaks R, Mattiello A, Bueno-de-Mesquita HB, Peeters PH, Travis RC, Petersson J, Hansson O, Arriola L, Jimenez-Martin JM, Tjonneland A, Halkjaer J, Agnoli C, Sacerdote C, Bonet C, Trichopoulou A, Gavrila D, Overvad K, Weiderpass E, Palli D, Quiros JR, Tumino R, Khaw KT, Wareham N, Barricante-Gurrea A, Fedirko V, Ferrari P, Clavel-Chapelon F, Boutron-Ruault MC, Boeing H, Vigl M, Middleton L, Riboli E, Vineis P (2013) Prediagnostic body fat and risk of death from amyotrophic lateral sclerosis: the EPIC cohort. Neurology 80(9):829–838. 10.1212/WNL.0b013e3182840689 23390184 10.1212/WNL.0b013e3182840689 PMC3598455

[29] Garcia H, Song M (2019) Early-life obesity and adulthood colorectal cancer risk: a meta-analysis. Rev Panam Salud Publica 43:e3. 10.26633/RPSP.2019.3 31093227 10.26633/RPSP.2019.3 PMC6393738

[30] Garg R, Rustagi T (2018) Management of hypertriglyceridemia induced acute pancreatitis. Biomed Res Int 2018:4721357. 10.1155/2018/4721357 30148167 10.1155/2018/4721357 PMC6083537

[31] Gilliland FD, Berhane K, Islam T, McConnell R, Gauderman WJ, Gilliland SS, Avol E, Peters JM (2003) Obesity and the risk of newly diagnosed asthma in school-age children. Am J Epidemiol 158(5):406–415. 10.1093/aje/kwg175 12936895 10.1093/aje/kwg175

[32] Girman CJ, Kou TD, Cai B, Alexander CM, O’Neill EA, Williams-Herman DE, Katz L (2010) Patients with type 2 diabetes mellitus have higher risk for acute pancreatitis compared with those without diabetes. Diabetes Obes Metab 12(9):766–771. 10.1111/j.1463-1326.2010.01231.x 20649628 10.1111/j.1463-1326.2010.01231.x

[33] Global Burden of Disease Cancer Collaboration, Fitzmaurice C, Allen C, Barber RM, Barregard L, Bhutta ZA, Brenner H, Dicker DJ, Chimed-Orchir O, Dandona R, Dandona L, Fleming T, Forouzanfar MH, Hancock J, Hay RJ, Hunter-Merrill R, Huynh C, Hosgood HD, Johnson CO, Jonas JB, Khubchandani J, Kumar GA, Kutz M, Lan Q, Larson HJ, Liang X, Lim SS, Lopez AD, MacIntyre MF, Marczak L, Marquez N, Mokdad AH, Pinho C, Pourmalek F, Salomon JA, Sanabria JR, Sandar L, Sartorius B, Schwartz SM, Shackelford KA, Shibuya K, Stanaway J, Steiner C, Sun J, Takahashi K, Vollset SE, Vos T, Wagner JA, Wang H, Westerman R, Zeeb H, Zoeckler L, Abd-Allah F, Ahmed MB, Alabed S, Alam NK, Aldhahri SF, Alem G, Alemayohu MA, Ali R, Al-Raddadi R, Amare A, Amoako Y, Artaman A, Asayesh H, Atnafu N, Awasthi A, Saleem HB, Barac A, Bedi N, Bensenor I, Berhane A, Bernabe E, Betsu B, Binagwaho A, Boneya D, Campos-Nonato I, Castaneda-Orjuela C, Catala-Lopez F, Chiang P, Chibueze C, Chitheer A, Choi JY, Cowie B, Damtew S, das Neves J, Dey S, Dharmaratne S, Dhillon P, Ding E, Driscoll T, Ekwueme D, Endries AY, Farvid M, Farzadfar F, Fernandes J, Fischer F, TT GH, Gebru A, Gopalani S, Hailu A, Horino M, Horita N, Husseini A, Huybrechts I, Inoue M, Islami F, Jakovljevic M, James S, Javanbakht M, Jee SH, Kasaeian A, Kedir MS, Khader YS, Khang YH, Kim D, Leigh J, Linn S, Lunevicius R, El Razek HMA, Malekzadeh R, Malta DC, Marcenes W, Markos D, Melaku YA, Meles KG, Mendoza W, Mengiste DT, Meretoja TJ, Miller TR, Mohammad KA, Mohammadi A, Mohammed S, Moradi-Lakeh M, Nagel G, Nand D, Le Nguyen Q, Nolte S, Ogbo FA, Oladimeji KE, Oren E, Pa M, Park EK, Pereira DM, Plass D, Qorbani M, Radfar A, Rafay A, Rahman M, Rana SM, Soreide K, Satpathy M, Sawhney M, Sepanlou SG, Shaikh MA, She J, Shiue I, Shore HR, Shrime MG, So S, Soneji S, Stathopoulou V, Stroumpoulis K, Sufiyan MB, Sykes BL, Tabares-Seisdedos R, Tadese F, Tedla BA, Tessema GA, Thakur JS, Tran BX, Ukwaja KN, Uzochukwu BSC, Vlassov VV, Weiderpass E, Wubshet Terefe M, Yebyo HG, Yimam HH, Yonemoto N, Younis MZ, Yu C, Zaidi Z, Zaki MES, Zenebe ZM, Murray CJL, Naghavi M (2017) Global, regional, and national cancer incidence, mortality, years of life lost, years lived with disability, and disability-adjusted life-years for 32 cancer groups, 1990 to 2015: a systematic analysis for the Global Burden of Disease Study. JAMA Oncol 3(4):524–548. 10.1001/jamaoncol.2016.5688 27918777 10.1001/jamaoncol.2016.5688 PMC6103527

[34] Global Burden of Disease Collaborative Network (2020) Global Burden of Disease Study 2019 (GBD 2019). Institute for Health Metrics and Evaluation (IHME). https://www.healthdata.org/research-analysis/gbd Accessed 11 Dec 2024

[35] Gold DR, Damokosh AI, Dockery DW, Berkey CS (2003) Body-mass index as a predictor of incident asthma in a prospective cohort of children. Pediatr Pulmonol 36(6):514–521. 10.1002/ppul.10376 14618644 10.1002/ppul.10376

[36] Golledge J, Clancy P, Jamrozik K, Norman PE (2007) Obesity, adipokines, and abdominal aortic aneurysm: health in men study. Circulation 116(20):2275–2279. 10.1161/CIRCULATIONAHA.107.717926 17967974 10.1161/CIRCULATIONAHA.107.717926

[37] Gonzalez MC, Pastore CA, Orlandi SP, Heymsfield SB (2014) Obesity paradox in cancer: new insights provided by body composition. Am J Clin Nutr 99(5):999–1005. 10.3945/ajcn.113.071399 24572565 10.3945/ajcn.113.071399

[38] Grodin JM, Siiteri PK, MacDonald PC (1973) Source of estrogen production in postmenopausal women. J Clin Endocrinol Metab 36(2):207–214. 10.1210/jcem-36-2-207 4688315 10.1210/jcem-36-2-207

[39] Gustafson D, Rothenberg E, Blennow K, Steen B, Skoog I (2003) An 18-year follow-up of overweight and risk of Alzheimer disease. Arch Intern Med 163(13):1524–1528. 10.1001/archinte.163.13.1524 12860573 10.1001/archinte.163.13.1524

[40] Hall JE, do Carmo JM, da Silva AA, Wang Z, Hall ME (2019) Obesity, kidney dysfunction and hypertension: mechanistic links. Nat Rev Nephrol 15(6):367–385. 10.1038/s41581-019-0145-4 31015582 10.1038/s41581-019-0145-4 PMC7278043

[41] Hass DJ, Brensinger CM, Lewis JD, Lichtenstein GR (2006) The impact of increased body mass index on the clinical course of Crohn’s disease. Clin Gastroenterol Hepatol 4(4):482–488. 10.1016/j.cgh.2005.12.015 16616354 10.1016/j.cgh.2005.12.015

[42] Hedstrom AK, Olsson T, Alfredsson L (2012) High body mass index before age 20 is associated with increased risk for multiple sclerosis in both men and women. Mult Scler 18(9):1334–1336. 10.1177/1352458512436596 22328681 10.1177/1352458512436596

[43] Hemminki K, Li X, Sundquist J, Sundquist K (2012) Risk of asthma and autoimmune diseases and related conditions in patients hospitalized for obesity. Ann Med 44(3):289–295. 10.3109/07853890.2010.547515 21284531 10.3109/07853890.2010.547515

[44] Henning RJ (2021) Obesity and obesity-induced inflammatory disease contribute to atherosclerosis: a review of the pathophysiology and treatment of obesity. Am J Cardiovasc Dis 11(4):504–529 34548951 PMC8449192

[45] Hines RB, Shanmugam C, Waterbor JW, McGwin G Jr, Funkhouser E, Coffey CS, Posey J, Manne U (2009) Effect of comorbidity and body mass index on the survival of African-American and Caucasian patients with colon cancer. Cancer 115(24):5798–5806. 10.1002/cncr.24598 19937953 10.1002/cncr.24598 PMC2795032

[46] Hoyo C, Cook MB, Kamangar F, Freedman ND, Whiteman DC, Bernstein L, Brown LM, Risch HA, Ye W, Sharp L, Wu AH, Ward MH, Casson AG, Murray LJ, Corley DA, Nyren O, Pandeya N, Vaughan TL, Chow WH, Gammon MD (2012) Body mass index in relation to oesophageal and oesophagogastric junction adenocarcinomas: a pooled analysis from the International BEACON Consortium. Int J Epidemiol 41(6):1706–1718. 10.1093/ije/dys176 23148106 10.1093/ije/dys176 PMC3535758

[47] Huang W, Xiao Y, Zhang L, Liu H (2024) Association between a body shape index and Parkinson’s disease: a large cross-sectional study from NHANES. Heliyon 10(4):e26557. 10.1016/j.heliyon.2024.e26557 38420444 10.1016/j.heliyon.2024.e26557 PMC10900994

[48] Ilic M, Ilic I (2016) Epidemiology of pancreatic cancer. World J Gastroenterol 22(44):9694–9705. 10.3748/wjg.v22.i44.9694 27956793 10.3748/wjg.v22.i44.9694 PMC5124974

[49] Janse van Mantgem MR, van Eijk RPA, van der Burgh HK, Tan HHG, Westeneng HJ, van Es MA, Veldink JH, van den Berg LH (2020) Prognostic value of weight loss in patients with amyotrophic lateral sclerosis: a population-based study. J Neurol Neurosurg Psychiatry 91(8):867–875. 10.1136/jnnp-2020-322909 32576612 10.1136/jnnp-2020-322909

[50] Kaltoft M, Langsted A, Nordestgaard BG (2020) Obesity as a causal risk factor for aortic valve stenosis. J Am Coll Cardiol 75(2):163–176. 10.1016/j.jacc.2019.10.050 31948645 10.1016/j.jacc.2019.10.050

[51] Kannel WB, Brand N, Skinner JJ Jr, Dawber TR, McNamara PM (1967) The relation of adiposity to blood pressure and development of hypertension. The Framingham study. Ann Intern Med 67(1):48–59. 10.7326/0003-4819-67-1-48 6028658 10.7326/0003-4819-67-1-48

[52] Khalili H, Ananthakrishnan AN, Konijeti GG, Higuchi LM, Fuchs CS, Richter JM, Chan AT (2015) Measures of obesity and risk of Crohn’s disease and ulcerative colitis. Inflamm Bowel Dis 21(2):361–368. 10.1097/MIB.0000000000000283 25563694 10.1097/MIB.0000000000000283 PMC4308549

[53] Khan MAB, Hashim MJ, King JK, Govender RD, Mustafa H, Al Kaabi J (2020) Epidemiology of type 2 diabetes - global burden of disease and forecasted trends. J Epidemiol Glob Health 10(1):107–111. 10.2991/jegh.k.191028.001 32175717 10.2991/jegh.k.191028.001 PMC7310804

[54] Khatua B, El-Kurdi B, Singh VP (2017) Obesity and pancreatitis. Curr Opin Gastroenterol 33(5):374–382. 10.1097/MOG.0000000000000386 28719397 10.1097/MOG.0000000000000386 PMC6640854

[55] Kim JA, Montagnani M, Chandrasekran S, Quon MJ (2012) Role of lipotoxicity in endothelial dysfunction. Heart Fail Clin 8(4):589–607. 10.1016/j.hfc.2012.06.012 22999242 10.1016/j.hfc.2012.06.012 PMC4126197

[56] Kim JH, Oh CM, Yoo JH (2023) Obesity and novel management of inflammatory bowel disease. World J Gastroenterol 29(12):1779–1794. 10.3748/wjg.v29.i12.1779 37032724 10.3748/wjg.v29.i12.1779 PMC10080699

[57] Klein S, Gastaldelli A, Yki-Jarvinen H, Scherer PE (2022) Why does obesity cause diabetes? Cell Metab 34(1):11–20. 10.1016/j.cmet.2021.12.012 34986330 10.1016/j.cmet.2021.12.012 PMC8740746

[58] Klop B, Elte JW, Cabezas MC (2013) Dyslipidemia in obesity: mechanisms and potential targets. Nutrients 5(4):1218–1240. 10.3390/nu5041218 23584084 10.3390/nu5041218 PMC3705344

[59] Larsson SC, Orsini N, Wolk A (2007) Body mass index and pancreatic cancer risk: a meta-analysis of prospective studies. Int J Cancer 120(9):1993–1998. 10.1002/ijc.22535 17266034 10.1002/ijc.22535

[60] Larsson SC, Wolk A, Hakansson N, Back M (2017) Overall and abdominal obesity and incident aortic valve stenosis: two prospective cohort studies. Eur Heart J 38(28):2192–2197. 10.1093/eurheartj/ehx140 28402538 10.1093/eurheartj/ehx140 PMC5837465

[61] Lauby-Secretan B, Scoccianti C, Loomis D, Grosse Y, Bianchini F, Straif K, International Agency for Research on Cancer Handbook Working G (2016) Body fatness and cancer–viewpoint of the IARC Working Group. N Engl J Med 375(8):794–8. 10.1056/NEJMsr1606602 27557308 10.1056/NEJMsr1606602 PMC6754861

[62] Lavie CJ, Osman AF, Milani RV, Mehra MR (2003) Body composition and prognosis in chronic systolic heart failure: the obesity paradox. Am J Cardiol 91(7):891–894. 10.1016/s0002-9149(03)00031-6 12667583 10.1016/s0002-9149(03)00031-6

[63] Lavie CJ, De Schutter A, Parto P, Jahangir E, Kokkinos P, Ortega FB, Arena R, Milani RV (2016) Obesity and prevalence of cardiovascular diseases and prognosis-the obesity paradox updated. Prog Cardiovasc Dis 58(5):537–547. 10.1016/j.pcad.2016.01.008 26826295 10.1016/j.pcad.2016.01.008

[64] Lee K, Kruper L, Dieli-Conwright CM, Mortimer JE (2019) The impact of obesity on breast cancer diagnosis and treatment. Curr Oncol Rep 21(5):41. 10.1007/s11912-019-0787-1 30919143 10.1007/s11912-019-0787-1 PMC6437123

[65] Lempesis IG, Varrias D, Sagris M, Attaran RR, Altin ES, Bakoyiannis C, Palaiodimos L, Dalamaga M, Kokkinidis DG (2023) Obesity and peripheral artery disease: current evidence and controversies. Curr Obes Rep 12(3):264–279. 10.1007/s13679-023-00510-7 37243875 10.1007/s13679-023-00510-7 PMC10220347

[66] Lennon H, Sperrin M, Badrick E, Renehan AG (2016) The obesity paradox in cancer: a review. Curr Oncol Rep 18(9):56. 10.1007/s11912-016-0539-4 27475805 10.1007/s11912-016-0539-4 PMC4967417

[67] Li JY, Sun XH, Cai ZY, Shen DC, Yang XZ, Liu MS, Cui LY (2022) Correlation of weight and body composition with disease progression rate in patients with amyotrophic lateral sclerosis. Sci Rep 12(1):13292. 10.1038/s41598-022-16229-9 35918363 10.1038/s41598-022-16229-9 PMC9345931

[68] Li Y, Yang P, Ye J, Xu Q, Wu J, Wang Y (2024) Updated mechanisms of MASLD pathogenesis. Lipids Health Dis 23(1):117. 10.1186/s12944-024-02108-x 38649999 10.1186/s12944-024-02108-x PMC11034170

[69] Logroscino G, Sesso HD, Paffenbarger RS Jr, Lee IM (2007) Body mass index and risk of Parkinson’s disease: a prospective cohort study. Am J Epidemiol 166(10):1186–1190. 10.1093/aje/kwm211 17709328 10.1093/aje/kwm211

[70] Lutfullin I, Eveslage M, Bittner S, Antony G, Flaskamp M, Luessi F, Salmen A, Gisevius B, Klotz L, Korsukewitz C, Berthele A, Groppa S, Then Bergh F, Wildemann B, Bayas A, Tumani H, Meuth SG, Trebst C, Zettl UK, Paul F, Heesen C, Kuempfel T, Gold R, Hemmer B, Zipp F, Wiendl H, Lunemann JD, German Competence Network Multiple S (2023) Association of obesity with disease outcome in multiple sclerosis. J Neurol Neurosurg Psychiatry 94(1):57–61. 10.1136/jnnp-2022-329685 36319190 10.1136/jnnp-2022-329685 PMC9763191

[71] Magnus MC, Olsen SF, Granstrom C, Lund-Blix NA, Svensson J, Johannesen J, Fraser A, Skrivarhaug T, Joner G, Njolstad PR, Stordal K, Stene LC (2018) Paternal and maternal obesity but not gestational weight gain is associated with type 1 diabetes. Int J Epidemiol 47(2):417–426. 10.1093/ije/dyx266 29415279 10.1093/ije/dyx266 PMC5913633

[72] Mandic M, Li H, Safizadeh F, Niedermaier T, Hoffmeister M, Brenner H (2023) Is the association of overweight and obesity with colorectal cancer underestimated? An umbrella review of systematic reviews and meta-analyses. Eur J Epidemiol 38(2):135–144. 10.1007/s10654-022-00954-6 36680645 10.1007/s10654-022-00954-6 PMC9905196

[73] Manson JE, Colditz GA, Stampfer MJ, Willett WC, Rosner B, Monson RR, Speizer FE, Hennekens CH (1990) A prospective study of obesity and risk of coronary heart disease in women. N Engl J Med 322(13):882–889. 10.1056/NEJM199003293221303 2314422 10.1056/NEJM199003293221303

[74] Marcaccio CL, Schermerhorn ML (2021) Epidemiology of abdominal aortic aneurysms. Semin Vasc Surg 34(1):29–37. 10.1053/j.semvascsurg.2021.02.004 33757632 10.1053/j.semvascsurg.2021.02.004

[75] Misicka E, Gunzler D, Albert J, Briggs FBS (2023) Characterizing causal relationships of visceral fat and body shape on multiple sclerosis risk. Mult Scler Relat Disord 79:104964. 10.1016/j.msard.2023.104964 37659350 10.1016/j.msard.2023.104964 PMC10873055

[76] Mohamed-Ali V, Goodrick S, Rawesh A, Katz DR, Miles JM, Yudkin JS, Klein S, Coppack SW (1997) Subcutaneous adipose tissue releases interleukin-6, but not tumor necrosis factor-alpha, in vivo. J Clin Endocrinol Metab 82(12):4196–4200. 10.1210/jcem.82.12.4450 9398739 10.1210/jcem.82.12.4450

[77] Munger KL, Chitnis T, Ascherio A (2009) Body size and risk of MS in two cohorts of US women. Neurology 73(19):1543–1550. 10.1212/WNL.0b013e3181c0d6e0 19901245 10.1212/WNL.0b013e3181c0d6e0 PMC2777074

[78] Munger KL, Bentzen J, Laursen B, Stenager E, Koch-Henriksen N, Sorensen TI, Baker JL (2013) Childhood body mass index and multiple sclerosis risk: a long-term cohort study. Mult Scler 19(10):1323–1329. 10.1177/1352458513483889 23549432 10.1177/1352458513483889 PMC4418015

[79] Nakayama Y, Shimizu T, Matsuda C, Haraguchi M, Hayashi K, Bokuda K, Nagao M, Kawata A, Ishikawa-Takata K, Isozaki E (2019) Body weight variation predicts disease progression after invasive ventilation in amyotrophic lateral sclerosis. Sci Rep 9(1):12262. 10.1038/s41598-019-48831-9 31439899 10.1038/s41598-019-48831-9 PMC6706382

[80] Narayan KM, Boyle JP, Thompson TJ, Gregg EW, Williamson DF (2007) Effect of BMI on lifetime risk for diabetes in the U.S. Diabetes Care 30(6):1562–6. 10.2337/dc06-2544 17372155 10.2337/dc06-2544

[81] Ni Mhurchu C, Rodgers A, Pan WH, Gu DF, Woodward M, Asia Pacific Cohort Studies C (2004) Body mass index and cardiovascular disease in the Asia-Pacific Region: an overview of 33 cohorts involving 310 000 participants. Int J Epidemiol 33(4):751–758. 10.1093/ije/dyh163 15105409 10.1093/ije/dyh163

[82] Nourhashemi F, Deschamps V, Larrieu S, Letenneur L, Dartigues JF, Barberger-Gateau P, PsPA Q (2003) Body mass index and incidence of dementia: the PAQUID study. Neurology 60(1):117–119. 10.1212/01.wnl.0000038910.46217.aa 12525731 10.1212/01.wnl.0000038910.46217.aa

[83] O’Brien PD, Hinder LM, Callaghan BC, Feldman EL (2017) Neurological consequences of obesity. Lancet Neurol 16(6):465–477. 10.1016/S1474-4422(17)30084-4 28504110 10.1016/S1474-4422(17)30084-4 PMC5657398

[84] Olsen CM, Green AC, Whiteman DC, Sadeghi S, Kolahdooz F, Webb PM (2007) Obesity and the risk of epithelial ovarian cancer: a systematic review and meta-analysis. Eur J Cancer 43(4):690–709. 10.1016/j.ejca.2006.11.010 17223544 10.1016/j.ejca.2006.11.010

[85] Ong JP, Elariny H, Collantes R, Younoszai A, Chandhoke V, Reines HD, Goodman Z, Younossi ZM (2005) Predictors of nonalcoholic steatohepatitis and advanced fibrosis in morbidly obese patients. Obes Surg 15(3):310–315. 10.1381/0960892053576820 15826462 10.1381/0960892053576820

[86] Onstad MA, Schmandt RE, Lu KH (2016) Addressing the role of obesity in endometrial cancer risk, prevention, and treatment. J Clin Oncol 34(35):4225–4230. 10.1200/JCO.2016.69.4638 27903150 10.1200/JCO.2016.69.4638 PMC5455320

[87] Osnabrugge RL, Mylotte D, Head SJ, Van Mieghem NM, Nkomo VT, LeReun CM, Bogers AJ, Piazza N, Kappetein AP (2013) Aortic stenosis in the elderly: disease prevalence and number of candidates for transcatheter aortic valve replacement: a meta-analysis and modeling study. J Am Coll Cardiol 62(11):1002–1012. 10.1016/j.jacc.2013.05.015 23727214 10.1016/j.jacc.2013.05.015

[88] Palacios N, Gao X, McCullough ML, Jacobs EJ, Patel AV, Mayo T, Schwarzschild MA, Ascherio A (2011) Obesity, diabetes, and risk of Parkinson’s disease. Mov Disord 26(12):2253–2259. 10.1002/mds.23855 21739472 10.1002/mds.23855 PMC3627531

[89] Park KY, Nam GE, Han K, Park HK, Hwang HS (2022) Waist circumference and risk of Parkinson’s disease. NPJ Parkinsons Dis 8(1):89. 10.1038/s41531-022-00353-4 35803940 10.1038/s41531-022-00353-4 PMC9270375

[90] Parra-Landazury NM, Cordova-Gallardo J, Mendez-Sanchez N (2021) Obesity and gallstones. Visc Med 37(5):394–402. 10.1159/000515545 34722722 10.1159/000515545 PMC8543292

[91] Pati S, Irfan W, Jameel A, Ahmed S, Shahid RK (2023) Obesity and cancer: a current overview of epidemiology, pathogenesis, outcomes, and management. Cancers (Basel) 15(2). 10.3390/cancers15020485 10.3390/cancers15020485 PMC9857053 36672434

[92] Peters MC, McGrath KW, Hawkins GA, Hastie AT, Levy BD, Israel E, Phillips BR, Mauger DT, Comhair SA, Erzurum SC, Johansson MW, Jarjour NN, Coverstone AM, Castro M, Holguin F, Wenzel SE, Woodruff PG, Bleecker ER, Fahy JV, National Heart L, and Blood Institute Severe Asthma Research P (2016) Plasma interleukin-6 concentrations, metabolic dysfunction, and asthma severity: a cross-sectional analysis of two cohorts. Lancet Respir Med 4(7):574–584. 10.1016/S2213-2600(16)30048-0 27283230 10.1016/S2213-2600(16)30048-0 PMC5007068

[93] Peters U, Dixon AE, Forno E (2018) Obesity and asthma. J Allergy Clin Immunol 141(4):1169–1179. 10.1016/j.jaci.2018.02.004 29627041 10.1016/j.jaci.2018.02.004 PMC5973542

[94] Picon-Ruiz M, Morata-Tarifa C, Valle-Goffin JJ, Friedman ER, Slingerland JM (2017) Obesity and adverse breast cancer risk and outcome: mechanistic insights and strategies for intervention. CA Cancer J Clin 67(5):378–397. 10.3322/caac.21405 28763097 10.3322/caac.21405 PMC5591063

[95] Povova J, Ambroz P, Bar M, Pavukova V, Sery O, Tomaskova H, Janout V (2012) Epidemiological of and risk factors for Alzheimer’s disease: a review. Biomed Pap Med Fac Univ Palacky Olomouc Czech Repub 156(2):108–114. 10.5507/bp.2012.055 22837131 10.5507/bp.2012.055

[96] Profenno LA, Porsteinsson AP, Faraone SV (2010) Meta-analysis of Alzheimer’s disease risk with obesity, diabetes, and related disorders. Biol Psychiatry 67(6):505–512. 10.1016/j.biopsych.2009.02.013 19358976 10.1016/j.biopsych.2009.02.013

[97] Quek J, Chan KE, Wong ZY, Tan C, Tan B, Lim WH, Tan DJH, Tang ASP, Tay P, Xiao J, Yong JN, Zeng RW, Chew NWS, Nah B, Kulkarni A, Siddiqui MS, Dan YY, Wong VW, Sanyal AJ, Noureddin M, Muthiah M, Ng CH (2023) Global prevalence of non-alcoholic fatty liver disease and non-alcoholic steatohepatitis in the overweight and obese population: a systematic review and meta-analysis. Lancet Gastroenterol Hepatol 8(1):20–30. 10.1016/S2468-1253(22)00317-X 36400097 10.1016/S2468-1253(22)00317-X

[98] Renehan AG, Tyson M, Egger M, Heller RF, Zwahlen M (2008) Body-mass index and incidence of cancer: a systematic review and meta-analysis of prospective observational studies. Lancet 371(9612):569–578. 10.1016/S0140-6736(08)60269-X 18280327 10.1016/S0140-6736(08)60269-X

[99] Riccardi G, Giacco R, Rivellese AA (2004) Dietary fat, insulin sensitivity and the metabolic syndrome. Clin Nutr 23(4):447–456. 10.1016/j.clnu.2004.02.006 15297079 10.1016/j.clnu.2004.02.006

[100] Rocha VZ, Libby P (2009) Obesity, inflammation, and atherosclerosis. Nat Rev Cardiol 6(6):399–409. 10.1038/nrcardio.2009.55 19399028 10.1038/nrcardio.2009.55

[101] Roos E, Grotta A, Yang F, Bellocco R, Ye W, Adami HO, Wirdefeldt K, Trolle Lagerros Y (2018) Body mass index, sitting time, and risk of Parkinson disease. Neurology 90(16):e1413–e1417. 10.1212/WNL.0000000000005328 29661903 10.1212/WNL.0000000000005328 PMC5902782

[102] Rovella V, Anemona L, Cardellini M, Scimeca M, Saggini A, Santeusanio G, Bonanno E, Montanaro M, Legramante IM, Ippoliti A, Di Daniele N, Federici M, Mauriello A (2018) The role of obesity in carotid plaque instability: interaction with age, gender, and cardiovascular risk factors. Cardiovasc Diabetol 17(1):46. 10.1186/s12933-018-0685-0 29598820 10.1186/s12933-018-0685-0 PMC5874994

[103] Rustgi VK, Li Y, Gupta K, Minacapelli CD, Bhurwal A, Catalano C, Elsaid MI (2021) Bariatric surgery reduces cancer risk in adults with nonalcoholic fatty liver disease and severe obesity. Gastroenterology 161(1):171-184 e10. 10.1053/j.gastro.2021.03.021 33744305 10.1053/j.gastro.2021.03.021

[104] Sand FL, Urbute A, Ring LL, Kjaer AK, Belmonte F, Kjaer SK (2023) The influence of overweight and obesity on participation in cervical cancer screening: a systematic review and meta-analysis. Prev Med 172:107519. 10.1016/j.ypmed.2023.107519 37080502 10.1016/j.ypmed.2023.107519

[105] Schlesinger S, Siegert S, Koch M, Walter J, Heits N, Hinz S, Jacobs G, Hampe J, Schafmayer C, Nothlings U (2014) Postdiagnosis body mass index and risk of mortality in colorectal cancer survivors: a prospective study and meta-analysis. Cancer Causes Control 25(10):1407–1418. 10.1007/s10552-014-0435-x 25037235 10.1007/s10552-014-0435-x

[106] Schlesinger S, Aleksandrova K, Abar L, Vieria AR, Vingeliene S, Polemiti E, Stevens CAT, Greenwood DC, Chan DSM, Aune D, Norat T (2017) Adult weight gain and colorectal adenomas-a systematic review and meta-analysis. Ann Oncol 28(6):1217–1229. 10.1093/annonc/mdx080 28327995 10.1093/annonc/mdx080

[107] Shahid RK, Ahmed S, Le D, Yadav S (2021) Diabetes and cancer: risk, challenges, management and outcomes. Cancers (Basel) 13(22). 10.3390/cancers13225735 10.3390/cancers13225735 PMC8616213 34830886

[108] Shariq OA, McKenzie TJ (2020) Obesity-related hypertension: a review of pathophysiology, management, and the role of metabolic surgery. Gland Surg 9(1):80–93. 10.3390/cancers13225735 32206601 10.21037/gs.2019.12.03 PMC7082272

[109] Shaw E, Farris M, McNeil J, Friedenreich C (2016) Obesity and endometrial cancer. Recent Results Cancer Res 208:107–136. 10.1007/978-3-319-42542-9_7 27909905 10.1007/978-3-319-42542-9_7

[110] Shimizu T, Kimura N, Mieno M, Hori D, Shiraishi M, Tashima Y, Yuri K, Itagaki R, Aizawa K, Kawahito K, Yamaguchi A (2020) Effects of obesity on outcomes of acute type A aortic dissection repair in Japan. Circ Rep 2(11):639–647. 10.1253/circrep.CR-20-0098 33693190 10.1253/circrep.CR-20-0098 PMC7937495

[111] Sideleva O, Suratt BT, Black KE, Tharp WG, Pratley RE, Forgione P, Dienz O, Irvin CG, Dixon AE (2012) Obesity and asthma: an inflammatory disease of adipose tissue not the airway. Am J Respir Crit Care Med 186(7):598–605. 10.1164/rccm.201203-0573OC 22837379 10.1164/rccm.201203-0573OC PMC3480522

[112] Smith E, Hay P, Campbell L, Trollor JN (2011) A review of the association between obesity and cognitive function across the lifespan: implications for novel approaches to prevention and treatment. Obes Rev 12(9):740–755. 10.1111/j.1467-789X.2011.00920.x 21991597 10.1111/j.1467-789X.2011.00920.x

[113] Sung H, Siegel RL, Torre LA, Pearson-Stuttard J, Islami F, Fedewa SA, Goding Sauer A, Shuval K, Gapstur SM, Jacobs EJ, Giovannucci EL, Jemal A (2019) Global patterns in excess body weight and the associated cancer burden. CA Cancer J Clin 69(2):88–112. 10.3322/caac.21499 30548482 10.3322/caac.21499

[114] Verges B (2015) Pathophysiology of diabetic dyslipidaemia: where are we? Diabetologia 58(5):886–899. 10.1007/s00125-015-3525-8 25725623 10.1007/s00125-015-3525-8 PMC4392164

[115] Wang GJ, Gao CF, Wei D, Wang C, Ding SQ (2009) Acute pancreatitis: etiology and common pathogenesis. World J Gastroenterol 15(12):1427–1430. 10.3748/wjg.15.1427 19322914 10.3748/wjg.15.1427 PMC2665136

[116] Wiese ML, Aghdassi AA, Lerch MM, Steveling A (2021) Excess body weight and pancreatic disease. Visc Med 37(4):281–286. 10.1159/000517147 34540944 10.1159/000517147 PMC8406348

[117] World Health Organization (2022) Invisible numbers: the true extent of noncommunicable diseases and what to do about them. https://apps.who.int/iris/rest/bitstreams/1466662/retrieve Accessed 11 Dec 2024

[118] World Health Organization (2024) Obesity and overweight fact sheet. https://www.who.int/news-room/fact-sheets/detail/obesity-and-overweight Accessed 11 Dec 2024

[119] Wu H, Ballantyne CM (2020) Metabolic inflammation and insulin resistance in obesity. Circ Res 126(11):1549–1564. 10.1161/CIRCRESAHA.119.315896 32437299 10.1161/CIRCRESAHA.119.315896 PMC7250139

[120] Ye P, Xi Y, Huang Z, Xu P (2020) Linking obesity with colorectal cancer: epidemiology and mechanistic insights. Cancers (Basel) 12(6). 10.3390/cancers12061408 10.3390/cancers12061408 PMC7352519 32486076

[121] Yetman AT, McCrindle BW (2010) The prevalence and clinical impact of obesity in adults with Marfan syndrome. Can J Cardiol 26(4):137–139. 10.1016/s0828-282x(10)70370-6 20386774 10.1016/s0828-282x(10)70370-6 PMC2886547

[122] Zhang Z, Lai HJ, Roberg KA, Gangnon RE, Evans MD, Anderson EL, Pappas TE, Dasilva DF, Tisler CJ, Salazar LP, Gern JE, Lemanske RF Jr (2010) Early childhood weight status in relation to asthma development in high-risk children. J Allergy Clin Immunol 126(6):1157–1162. 10.1016/j.jaci.2010.09.011 21051081 10.1016/j.jaci.2010.09.011 PMC2998556

[123] Zucker I, Zloof Y, Bardugo A, Tsur AM, Lutski M, Cohen Y, Cukierman-Yaffe T, Minsky N, Derazne E, Tzur D, Melzer Cohen C, Pinhas-Hamiel O, Chodick G, Raz I, Afek A, Gerstein HC, Tirosh A, Twig G (2022) Obesity in late adolescence and incident type 1 diabetes in young adulthood. Diabetologia 65(9):1473–1482. 10.1007/s00125-022-05722-5 35665825 10.1007/s00125-022-05722-5

