# Supplementary material for: The obesity pandemic and its impact on non-communicable disease burden
Source: Pflugers Arch. 2025 Feb 10;477(5):657–68. doi: 10.1007/s00424-025-03066-8 (PMC12003543; doi:10.1007/s00424-025-03066-8)
Supplement: Supplementary file 1 — Supplementary file1 (DOCX 187 kb) [file 424_2025_3066_MOESM1_ESM.docx]

Supplementary Table 1: NCDs and their relation to obesity.

| **Mental disorders** | Schizophrenia[94]  Depressive disorders[87] [106] [7]  Bipolar disorder[62] [117] [95]  Anxiety disorder[6]  Eating disorders[26]  Autism spectrum disorders[132] [70] [51]  Attention deficit/hyperactivity disorder[24] [108] [64] [82]  Conduct disorder[103]  Idiopathic developmental intellectual disorder* |
| --- | --- |
| **Substance abuse disorders** | Alcohol use disorders[123]  Drug use disorders[123] |
| **Diabetes and kidney diseases** | Diabetes mellitus[88] [151]  Chronic kidney disease[69]  Acute glomerulonephritis* |
| **Skin and subcutaneous diseases** | Dermatitis[148] [129]  Urticaria[73] [142] [145] [68]  Decubitus ulcer[3]  Psoriasis[59] [71]  Bacterial skin diseases[19]  Scabies[104]  Fungal skin diseases[52]  Viral skin diseases[63]  Acne vulgaris[39]  Alopecia areata[80]  Pruritus* |
| **Neurological disorders** | Alzheimer’s disease and other dementias[36]  Parkinson’s disease[1]  Idiopathic epilepsy[72]  Multiple sclerosis[96]  Motor neuron disease[38]  Headache disorders[13] |
| **Digestive diseases** | Cirrhosis and other chronic liver diseases[110] [41]  Upper digestive system diseases[18] [15]  Appendicitis*  Paralytic ileus and intestinal obstruction[97]  Inguinal, femoral, and abdominal hernia[146] [116] [83]  Inflammatory bowel disease[67]  Vascular intestinal disorders*  Gallbladder and biliary diseases[131]  Pancreatitis[65] |
| **Chronic respiratory diseases** | Chronic obstructive pulmonary disease[147] [47]  Pneumoconiosis*  Asthma[149] [40] [42] [35]  Interstitial lung disease and pulmonary sarcoidosis[45] [122] |
| **Cardiovascular diseases** | Rheumatic heart disease*  Peripheral artery disease[81]  Endocarditis[48]  Ischemic heart disease[89] [101] [8] [9] [17] [12]  Stroke[130] [53] [114] [17]  Hypertensive heart disease[46] [29]  Non-rheumatic valvular heart disease[77] [61]  Cardiomyopathy and myocarditis [4]  Atrial fibrillation and flutter[44] [56]  Aortic aneurysm[43] [126] [143] |
| **Musculoskeletal disorders** | Rheumatoid arthritis[25] [111]  Osteoarthritis[98]  Low back pain[28] [50] [22]  Neck pain[138]  Gout[140] [78] |
| **Sense organ diseases** | Blindness and vision loss[100]  Age-related and other hearing loss[54] |
| **Neoplasms** | Lip and oral cavity cancer[55] [115]  Larynx cancer[66]  Tracheal, bronchus, and lung cancer[102]  Malignant skin melanoma[31]  Non-melanoma skin cancer[150]  Breast cancer[109] [79]  Cervical cancer[23]  Uterine cancer[105] [125]  Ovarian cancer[34]  Prostate cancer[119] [136]  Testicular cancer[11] [2]  Nasopharynx cancer[32]  Kidney cancer[99] [86]  Bladder cancer[21] [20]  Brain and central nervous system cancer[124]  Thyroid cancer[37]  Mesothelioma*  Hodgkin lymphoma[93]  Non-Hodgkin lymphoma[75] [134]  Multiple myeloma[91]  Leukemia[112] [84] [90]  Esophageal cancer[137]  Stomach cancer[58]  Colon and rectum cancer[141]  Liver cancer[120] [127]  Gallbladder and biliary tract cancer[76] [85] [133] [139]  Pancreatic cancer[10] [74] [118] |
| **Other NCDs** | Congenital birth defects[57]  Urinary diseases and male infertility[14] [16] [30] [27] [121]  Gynecological diseases[107] [144]  Hemoglobinopathies and hemolytic anemias*  Endocrine, metabolic, blood, and immune disorders[33] [128] [113] [135]  Oral disorders[5] [92] [49]  Sudden infant death syndrome[60] |

Color code: Black: positive correlation with obesity; Red: negative correlation with obesity; Blue: conflicting data; Yellow: no correlation found.
*: No data found

1. Abbott RD, Ross GW, White LR, Nelson JS, Masaki KH, Tanner CM, Curb JD, Blanchette PL, Popper JS, and Petrovitch H (2002) Midlife adiposity and the future risk of Parkinson's disease. Neurology 59(7): 1051-7. 10.1212/wnl.59.7.1051

2. Alam SS, Cantwell MM, Cardwell CR, Cook MB, and Murray LJ (2010) Maternal body mass index and risk of testicular cancer in male offspring: a systematic review and meta-analysis. Cancer Epidemiol 34(5): 509-15. 10.1016/j.canep.2010.07.006

3. Alipoor E, Mehrdadi P, Yaseri M, and Hosseinzadeh-Attar MJ (2021) Association of overweight and obesity with the prevalence and incidence of pressure ulcers: A systematic review and meta-analysis. Clin Nutr 40(9): 5089-5098. 10.1016/j.clnu.2021.08.006

4. Alpert MA (2001) Obesity cardiomyopathy: pathophysiology and evolution of the clinical syndrome. Am J Med Sci 321(4): 225-36. 10.1097/00000441-200104000-00003

5. Alswat K, Mohamed WS, Wahab MA, and Aboelil AA (2016) The Association Between Body Mass Index and Dental Caries: Cross-Sectional Study. J Clin Med Res 8(2): 147-52. 10.14740/jocmr2433w

6. Amiri S and Behnezhad S (2019) Obesity and anxiety symptoms: a systematic review and meta-analysis. Neuropsychiatr 33(2): 72-89. 10.1007/s40211-019-0302-9

7. Anguita-Ruiz A, Zarza-Rebollo JA, Perez-Gutierrez AM, Molina E, Gutierrez B, Bellon JA, Moreno-Peral P, Conejo-Ceron S, Aiarzaguena JM, Ballesta-Rodriguez MI, Fernandez A, Fernandez-Alonso C, Martin-Perez C, Monton-Franco C, Rodriguez-Bayon A, Torres-Martos A, Lopez-Isac E, Cervilla J, and Rivera M (2022) Body mass index interacts with a genetic-risk score for depression increasing the risk of the disease in high-susceptibility individuals. Transl Psychiatry 12(1): 30. 10.1038/s41398-022-01783-7

8. Atique SM, Shadbolt B, Marley P, and Farshid A (2016) Association Between Body Mass Index and Age of Presentation With Symptomatic Coronary Artery Disease. Clin Cardiol 39(11): 653-657. 10.1002/clc.22576

9. Azab M, Al-Shudifat AE, Johannessen A, Al-Shdaifat A, Agraib LM, and Tayyem RF (2018) Are Risk Factors for Coronary Artery Disease Different in Persons With and Without Obesity? Metab Syndr Relat Disord 16(8): 440-445. 10.1089/met.2017.0152

10. Berrington de Gonzalez A, Sweetland S, and Spencer E (2003) A meta-analysis of obesity and the risk of pancreatic cancer. Br J Cancer 89(3): 519-23. 10.1038/sj.bjc.6601140

11. Bjorge T, Tretli S, Lie AK, and Engeland A (2006) The impact of height and body mass index on the risk of testicular cancer in 600,000 Norwegian men. Cancer Causes Control 17(7): 983-7. 10.1007/s10552-006-0032-8

12. Bogers RP, Bemelmans WJ, Hoogenveen RT, Boshuizen HC, Woodward M, Knekt P, van Dam RM, Hu FB, Visscher TL, Menotti A, Thorpe RJ, Jr., Jamrozik K, Calling S, Strand BH, Shipley MJ, and Investigators B-CC (2007) Association of overweight with increased risk of coronary heart disease partly independent of blood pressure and cholesterol levels: a meta-analysis of 21 cohort studies including more than 300 000 persons. Arch Intern Med 167(16): 1720-8. 10.1001/archinte.167.16.1720

13. Bond DS, Roth J, Nash JM, and Wing RR (2011) Migraine and obesity: epidemiology, possible mechanisms and the potential role of weight loss treatment. Obes Rev 12(5): e362-71. 10.1111/j.1467-789X.2010.00791.x

14. Boyd C, Wood K, Whitaker D, and Assimos DG (2018) The influence of metabolic syndrome and its components on the development of nephrolithiasis. Asian J Urol 5(4): 215-222. 10.1016/j.ajur.2018.06.002

15. Camilleri M, Malhi H, and Acosta A (2017) Gastrointestinal Complications of Obesity. Gastroenterology 152(7): 1656-1670. 10.1053/j.gastro.2016.12.052

16. Carbone A, Al Salhi Y, Tasca A, Palleschi G, Fuschi A, De Nunzio C, Bozzini G, Mazzaferro S, and Pastore AL (2018) Obesity and kidney stone disease: a systematic review. Minerva Urol Nefrol 70(4): 393-400. 10.23736/S0393-2249.18.03113-2

17. Censin JC, Peters SAE, Bovijn J, Ferreira T, Pulit SL, Magi R, Mahajan A, Holmes MV, and Lindgren CM (2019) Causal relationships between obesity and the leading causes of death in women and men. PLoS Genet 15(10): e1008405. 10.1371/journal.pgen.1008405

18. Chang P and Friedenberg F (2014) Obesity and GERD. Gastroenterol Clin North Am 43(1): 161-73. 10.1016/j.gtc.2013.11.009

19. Cheong HS, Chang Y, Joo EJ, Cho A, and Ryu S (2019) Metabolic Obesity Phenotypes and Risk of Cellulitis: A Cohort Study. J Clin Med 8(7). 10.3390/jcm8070953

20. Choi JB, Kim JH, Hong SH, Han KD, and Ha US (2019) Association of body mass index with bladder cancer risk in men depends on abdominal obesity. World J Urol 37(11): 2393-2400. 10.1007/s00345-019-02690-1

21. Choi JB, Lee EJ, Han KD, Hong SH, and Ha US (2018) Estimating the impact of body mass index on bladder cancer risk: Stratification by smoking status. Sci Rep 8(1): 947. 10.1038/s41598-018-19531-7

22. Chou L, Brady SRE, Urquhart DM, Teichtahl AJ, Cicuttini FM, Pasco JA, Brennan-Olsen SL, and Wluka AE (2016) The Association Between Obesity and Low Back Pain and Disability Is Affected by Mood Disorders: A Population-Based, Cross-Sectional Study of Men. Medicine (Baltimore) 95(15): e3367. 10.1097/MD.0000000000003367

23. Clarke MA, Fetterman B, Cheung LC, Wentzensen N, Gage JC, Katki HA, Befano B, Demarco M, Schussler J, Kinney WK, Raine-Bennett TR, Lorey TS, Poitras NE, Castle PE, and Schiffman M (2018) Epidemiologic Evidence That Excess Body Weight Increases Risk of Cervical Cancer by Decreased Detection of Precancer. J Clin Oncol 36(12): 1184-1191. 10.1200/JCO.2017.75.3442

24. Cortese S and Tessari L (2017) Attention-Deficit/Hyperactivity Disorder (ADHD) and Obesity: Update 2016. Curr Psychiatry Rep 19(1): 4. 10.1007/s11920-017-0754-1

25. Crowson CS, Matteson EL, Davis JM, 3rd, and Gabriel SE (2013) Contribution of obesity to the rise in incidence of rheumatoid arthritis. Arthritis Care Res (Hoboken) 65(1): 71-7. 10.1002/acr.21660

26. da Luz FQ, Hay P, Touyz S, and Sainsbury A (2018) Obesity with Comorbid Eating Disorders: Associated Health Risks and Treatment Approaches. Nutrients 10(7). 10.3390/nu10070829

27. Darand M, Salimi Z, Ghorbani M, Sadeghi N, Babaie S, and Hosseinzadeh M (2023) Obesity is associated with quality of sperm parameters in men with infertility: a cross-sectional study. Reprod Health 20(1): 134. 10.1186/s12978-023-01664-2

28. Dario AB, Ferreira ML, Refshauge KM, Lima TS, Ordonana JR, and Ferreira PH (2015) The relationship between obesity, low back pain, and lumbar disc degeneration when genetics and the environment are considered: a systematic review of twin studies. Spine J 15(5): 1106-17. 10.1016/j.spinee.2015.02.001

29. de Simone G, Mancusi C, Izzo R, Losi MA, and Aldo Ferrara L (2016) Obesity and hypertensive heart disease: focus on body composition and sex differences. Diabetol Metab Syndr 8: 79. 10.1186/s13098-016-0193-x

30. Du Plessis SS, Cabler S, McAlister DA, Sabanegh E, and Agarwal A (2010) The effect of obesity on sperm disorders and male infertility. Nat Rev Urol 7(3): 153-61. 10.1038/nrurol.2010.6

31. Dusingize JC, Olsen CM, An J, Pandeya N, Law MH, Thompson BS, Goldstein AM, Iles MM, Webb PM, Neale RE, Ong JS, MacGregor S, and Whiteman DC (2020) Body mass index and height and risk of cutaneous melanoma: Mendelian randomization analyses. Int J Epidemiol 49(4): 1236-1245. 10.1093/ije/dyaa009

32. Feng R, Chang ET, Liu Z, Liu Q, Cai Y, Zhang Z, Chen G, Huang QH, Xie SH, Cao SM, Zhang Y, Yun J, Jia WH, Zheng Y, Liao J, Chen Y, Lin L, Ernberg I, Huang G, Zeng Y, Zeng YX, Adami HO, and Ye W (2019) Body mass index, body shape, and risk of nasopharyngeal carcinoma: A population-based case-control study in Southern China. Cancer Med 8(4): 1835-1844. 10.1002/cam4.2027

33. Fernandez CJ, Chacko EC, and Pappachan JM (2019) Male Obesity-related Secondary Hypogonadism - Pathophysiology, Clinical Implications and Management. Eur Endocrinol 15(2): 83-90. 10.17925/EE.2019.15.2.83

34. Foong KW and Bolton H (2017) Obesity and ovarian cancer risk: A systematic review. Post Reprod Health 23(4): 183-198. 10.1177/2053369117709225

35. Forno E, Young OM, Kumar R, Simhan H, and Celedon JC (2014) Maternal obesity in pregnancy, gestational weight gain, and risk of childhood asthma. Pediatrics 134(2): e535-46. 10.1542/peds.2014-0439

36. Forny-Germano L, De Felice FG, and Vieira M (2018) The Role of Leptin and Adiponectin in Obesity-Associated Cognitive Decline and Alzheimer's Disease. Front Neurosci 12: 1027. 10.3389/fnins.2018.01027

37. Franchini F, Palatucci G, Colao A, Ungaro P, Macchia PE, and Nettore IC (2022) Obesity and Thyroid Cancer Risk: An Update. Int J Environ Res Public Health 19(3). 10.3390/ijerph19031116

38. Gallo V, Wark PA, Jenab M, Pearce N, Brayne C, Vermeulen R, Andersen PM, Hallmans G, Kyrozis A, Vanacore N, Vahdaninia M, Grote V, Kaaks R, Mattiello A, Bueno-de-Mesquita HB, Peeters PH, Travis RC, Petersson J, Hansson O, Arriola L, Jimenez-Martin JM, Tjonneland A, Halkjaer J, Agnoli C, Sacerdote C, Bonet C, Trichopoulou A, Gavrila D, Overvad K, Weiderpass E, Palli D, Quiros JR, Tumino R, Khaw KT, Wareham N, Barricante-Gurrea A, Fedirko V, Ferrari P, Clavel-Chapelon F, Boutron-Ruault MC, Boeing H, Vigl M, Middleton L, Riboli E, and Vineis P (2013) Prediagnostic body fat and risk of death from amyotrophic lateral sclerosis: the EPIC cohort. Neurology 80(9): 829-38. 10.1212/WNL.0b013e3182840689

39. Gayen R, Podder I, Chakraborty I, and Chowdhury SN (2021) Sex Hormones, Metabolic Status, and Obesity in Female Patients with Acne Vulgaris Along with Clinical Correlation: An Observational Cross-Sectional Study. Indian J Dermatol 66(1): 60-66. 10.4103/ijd.IJD_82_20

40. Gilliland FD, Berhane K, Islam T, McConnell R, Gauderman WJ, Gilliland SS, Avol E, and Peters JM (2003) Obesity and the risk of newly diagnosed asthma in school-age children. Am J Epidemiol 158(5): 406-15. 10.1093/aje/kwg175

41. Gofton C, Upendran Y, Zheng MH, and George J (2023) MAFLD: How is it different from NAFLD? Clin Mol Hepatol 29(Suppl): S17-S31. 10.3350/cmh.2022.0367

42. Gold DR, Damokosh AI, Dockery DW, and Berkey CS (2003) Body-mass index as a predictor of incident asthma in a prospective cohort of children. Pediatr Pulmonol 36(6): 514-21. 10.1002/ppul.10376

43. Golledge J, Clancy P, Jamrozik K, and Norman PE (2007) Obesity, adipokines, and abdominal aortic aneurysm: Health in Men study. Circulation 116(20): 2275-9. 10.1161/CIRCULATIONAHA.107.717926

44. Goudis CA, Korantzopoulos P, Ntalas IV, Kallergis EM, and Ketikoglou DG (2015) Obesity and atrial fibrillation: A comprehensive review of the pathophysiological mechanisms and links. J Cardiol 66(5): 361-9. 10.1016/j.jjcc.2015.04.002

45. Guo X, Sunil C, and Qian G (2021) Obesity and the Development of Lung Fibrosis. Front Pharmacol 12: 812166. 10.3389/fphar.2021.812166

46. Hall JE, do Carmo JM, da Silva AA, Wang Z, and Hall ME (2019) Obesity, kidney dysfunction and hypertension: mechanistic links. Nat Rev Nephrol 15(6): 367-385. 10.1038/s41581-019-0145-4

47. Hanson C, Rutten EP, Wouters EF, and Rennard S (2014) Influence of diet and obesity on COPD development and outcomes. Int J Chron Obstruct Pulmon Dis 9: 723-33. 10.2147/COPD.S50111

48. Harris CM, Albaeni A, Wright S, and Norris KC (2019) Obesity as a Risk Factor Among Hospitalized Patients with Infective Endocarditis. Open Forum Infect Dis 6(10): ofz390. 10.1093/ofid/ofz390

49. Hayashi M, Morino K, Harada K, Miyazawa I, Ishikawa M, Yasuda T, Iwakuma Y, Kazushi Y, Motonobu M, Hiroshi M, and Atsushi I (2022) Real-world evidence of the impact of obesity on residual teeth in the Japanese population: A cross-sectional study. PLoS One 17(9): e0274465. 10.1371/journal.pone.0274465

50. Heuch I, Heuch I, Hagen K, and Zwart JA (2013) Body mass index as a risk factor for developing chronic low back pain: a follow-up in the Nord-Trondelag Health Study. Spine (Phila Pa 1976) 38(2): 133-9. 10.1097/BRS.0b013e3182647af2

51. Hill AP, Zuckerman KE, and Fombonne E (2015) Obesity and Autism. Pediatrics 136(6): 1051-61. 10.1542/peds.2015-1437

52. Hirt PA, Castillo DE, Yosipovitch G, and Keri JE (2019) Skin changes in the obese patient. J Am Acad Dermatol 81(5): 1037-1057. 10.1016/j.jaad.2018.12.070

53. Horn JW, Feng T, Morkedal B, Strand LB, Horn J, Mukamal K, and Janszky I (2021) Obesity and Risk for First Ischemic Stroke Depends on Metabolic Syndrome: The HUNT Study. Stroke 52(11): 3555-3561. 10.1161/STROKEAHA.120.033016

54. Hu H, Tomita K, Kuwahara K, Yamamoto M, Uehara A, Kochi T, Eguchi M, Okazaki H, Hori A, Sasaki N, Ogasawara T, Honda T, Yamamoto S, Nakagawa T, Miyamoto T, Imai T, Nishihara A, Nagahama S, Murakami T, Shimizu M, Akter S, Kashino I, Yamaguchi M, Kabe I, Mizoue T, Sone T, Dohi S, and Japan Epidemiology Collaboration on Occupational Health Study G (2020) Obesity and risk of hearing loss: A prospective cohort study. Clin Nutr 39(3): 870-875. 10.1016/j.clnu.2019.03.020

55. Huang J, Chan SC, Ko S, Lok V, Zhang L, Lin X, Lucero-Prisno DE, 3rd, Xu W, Zheng ZJ, Elcarte E, Withers M, Wong MCS, and Ncd Global Health Research Group AoPRU (2023) Disease burden, risk factors, and trends of lip, oral cavity, pharyngeal cancers: A global analysis. Cancer Med 12(17): 18153-18164. 10.1002/cam4.6391

56. Huxley RR, Lopez FL, Folsom AR, Agarwal SK, Loehr LR, Soliman EZ, Maclehose R, Konety S, and Alonso A (2011) Absolute and attributable risks of atrial fibrillation in relation to optimal and borderline risk factors: the Atherosclerosis Risk in Communities (ARIC) study. Circulation 123(14): 1501-8. 10.1161/CIRCULATIONAHA.110.009035

57. Iessa N and Berard A (2015) Update on Prepregnancy Maternal Obesity: Birth Defects and Childhood Outcomes. J Pediatr Genet 4(2): 71-83. 10.1055/s-0035-1556739

58. Ilic M and Ilic I (2022) Epidemiology of stomach cancer. World J Gastroenterol 28(12): 1187-1203. 10.3748/wjg.v28.i12.1187

59. Jensen P and Skov L (2016) Psoriasis and Obesity. Dermatology 232(6): 633-639. 10.1159/000455840

60. Johansson S, Villamor E, Altman M, Bonamy AK, Granath F, and Cnattingius S (2014) Maternal overweight and obesity in early pregnancy and risk of infant mortality: a population based cohort study in Sweden. BMJ 349: g6572. 10.1136/bmj.g6572

61. Kaltoft M, Langsted A, and Nordestgaard BG (2020) Obesity as a Causal Risk Factor for Aortic Valve Stenosis. J Am Coll Cardiol 75(2): 163-176. 10.1016/j.jacc.2019.10.050

62. Kambey PA, Kodzo LD, Serojane F, and Oluwasola BJ (2023) The bi-directional association between bipolar disorder and obesity: Evidence from Meta and bioinformatics analysis. Int J Obes (Lond) 47(6): 443-452. 10.1038/s41366-023-01277-6

63. Karjala Z, Neal D, and Rohrer J (2011) Association between HSV1 seropositivity and obesity: data from the National Health and Nutritional Examination Survey, 2007-2008. PLoS One 6(5): e19092. 10.1371/journal.pone.0019092

64. Khalife N, Kantomaa M, Glover V, Tammelin T, Laitinen J, Ebeling H, Hurtig T, Jarvelin MR, and Rodriguez A (2014) Childhood attention-deficit/hyperactivity disorder symptoms are risk factors for obesity and physical inactivity in adolescence. J Am Acad Child Adolesc Psychiatry 53(4): 425-36. 10.1016/j.jaac.2014.01.009

65. Khatua B, El-Kurdi B, and Singh VP (2017) Obesity and pancreatitis. Curr Opin Gastroenterol 33(5): 374-382. 10.1097/MOG.0000000000000386

66. Kim HB, Kim GJ, Han KD, and Joo YH (2021) Changes in metabolic syndrome status and risk of laryngeal cancer: A nationwide cohort study. PLoS One 16(6): e0252872. 10.1371/journal.pone.0252872

67. Kim JH, Oh CM, and Yoo JH (2023) Obesity and novel management of inflammatory bowel disease. World J Gastroenterol 29(12): 1779-1794. 10.3748/wjg.v29.i12.1779

68. Kim YH, Do Han K, Bang CH, Lee JH, Lee JY, Park YG, and Park YM (2021) High waist circumference rather than high body mass index may be a predictive risk factor for the longer disease duration of chronic spontaneous urticaria. Sci Rep 11(1): 1875. 10.1038/s41598-021-81484-1

69. Kovesdy CP, Furth SL, Zoccali C, and World Kidney Day Steering C (2017) Obesity and Kidney Disease: Hidden Consequences of the Epidemic. Can J Kidney Health Dis 4: 2054358117698669. 10.1177/2054358117698669

70. Krakowiak P, Walker CK, Bremer AA, Baker AS, Ozonoff S, Hansen RL, and Hertz-Picciotto I (2012) Maternal metabolic conditions and risk for autism and other neurodevelopmental disorders. Pediatrics 129(5): e1121-8. 10.1542/peds.2011-2583

71. Kunz M, Simon JC, and Saalbach A (2019) Psoriasis: Obesity and Fatty Acids. Front Immunol 10: 1807. 10.3389/fimmu.2019.01807

72. Ladino LD, Hernandez-Ronquillo L, and Tellez-Zenteno JF (2014) Obesity and its association with generalised epilepsy, idiopathic syndrome, and family history of epilepsy. Epileptic Disord 16(3): 343-53. 10.1684/epd.2014.0677

73. Lapi F, Cassano N, Pegoraro V, Cataldo N, Heiman F, Cricelli I, Levi M, Colombo D, Zagni E, Cricelli C, and Vena GA (2016) Epidemiology of chronic spontaneous urticaria: results from a nationwide, population-based study in Italy. Br J Dermatol 174(5): 996-1004. 10.1111/bjd.14470

74. Larsson SC, Orsini N, and Wolk A (2007) Body mass index and pancreatic cancer risk: A meta-analysis of prospective studies. Int J Cancer 120(9): 1993-8. 10.1002/ijc.22535

75. Larsson SC and Wolk A (2007) Obesity and risk of non-Hodgkin's lymphoma: a meta-analysis. Int J Cancer 121(7): 1564-70. 10.1002/ijc.22762

76. Larsson SC and Wolk A (2007) Obesity and the risk of gallbladder cancer: a meta-analysis. Br J Cancer 96(9): 1457-61. 10.1038/sj.bjc.6603703

77. Larsson SC, Wolk A, Hakansson N, and Back M (2017) Overall and abdominal obesity and incident aortic valve stenosis: two prospective cohort studies. Eur Heart J 38(28): 2192-2197. 10.1093/eurheartj/ehx140

78. Lee J, Lee JY, Lee JH, Jung SM, Suh YS, Koh JH, Kwok SK, Ju JH, Park KS, and Park SH (2015) Visceral fat obesity is highly associated with primary gout in a metabolically obese but normal weighted population: a case control study. Arthritis Res Ther 17(1): 79. 10.1186/s13075-015-0593-6

79. Lee K, Kruper L, Dieli-Conwright CM, and Mortimer JE (2019) The Impact of Obesity on Breast Cancer Diagnosis and Treatment. Curr Oncol Rep 21(5): 41. 10.1007/s11912-019-0787-1

80. Lee YB and Lee WS (2022) Alopecia Areata and Body Mass Index: A Retrospective Analysis of 257 Cases. Ann Dermatol 34(4): 305-308. 10.5021/ad.20.084

81. Lempesis IG, Varrias D, Sagris M, Attaran RR, Altin ES, Bakoyiannis C, Palaiodimos L, Dalamaga M, and Kokkinidis DG (2023) Obesity and Peripheral Artery Disease: Current Evidence and Controversies. Curr Obes Rep 12(3): 264-279. 10.1007/s13679-023-00510-7

82. Li L, Lagerberg T, Chang Z, Cortese S, Rosenqvist MA, Almqvist C, D'Onofrio BM, Hegvik TA, Hartman C, Chen Q, and Larsson H (2020) Maternal pre-pregnancy overweight/obesity and the risk of attention-deficit/hyperactivity disorder in offspring: a systematic review, meta-analysis and quasi-experimental family-based study. Int J Epidemiol 49(3): 857-875. 10.1093/ije/dyaa040

83. Li Z, Xia L, Li X, Guan Y, He H, and Jin L (2023) Body mass index and the risk of abdominal hernia: a Mendelian randomization study. Hernia 27(2): 423-429. 10.1007/s10029-022-02703-w

84. Lichtman MA (2010) Obesity and the risk for a hematological malignancy: leukemia, lymphoma, or myeloma. Oncologist 15(10): 1083-101. 10.1634/theoncologist.2010-0206

85. Liu H, Zhang Y, Ai M, Wang J, Jin B, Teng Z, Wang Y, and Li L (2016) Body Mass Index Can Increase the Risk of Gallbladder Cancer: A Meta-Analysis of 14 Cohort Studies. Med Sci Monit Basic Res 22: 146-155. 10.12659/msmbr.901651

86. Liu X, Sun Q, Hou H, Zhu K, Wang Q, Liu H, Zhang Q, Ji L, and Li D (2018) The association between BMI and kidney cancer risk: An updated dose-response meta-analysis in accordance with PRISMA guideline. Medicine (Baltimore) 97(44): e12860. 10.1097/MD.0000000000012860

87. Luppino FS, de Wit LM, Bouvy PF, Stijnen T, Cuijpers P, Penninx BW, and Zitman FG (2010) Overweight, obesity, and depression: a systematic review and meta-analysis of longitudinal studies. Arch Gen Psychiatry 67(3): 220-9. 10.1001/archgenpsychiatry.2010.2

88. Magnus MC, Olsen SF, Granstrom C, Lund-Blix NA, Svensson J, Johannesen J, Fraser A, Skrivarhaug T, Joner G, Njolstad PR, Stordal K, and Stene LC (2018) Paternal and maternal obesity but not gestational weight gain is associated with type 1 diabetes. Int J Epidemiol 47(2): 417-426. 10.1093/ije/dyx266

89. Manson JE, Colditz GA, Stampfer MJ, Willett WC, Rosner B, Monson RR, Speizer FE, and Hennekens CH (1990) A prospective study of obesity and risk of coronary heart disease in women. N Engl J Med 322(13): 882-9. 10.1056/NEJM199003293221303

90. Marley AR, Ryder JR, Turcotte LM, and Spector LG (2022) Maternal obesity and acute lymphoblastic leukemia risk in offspring: A summary of trends, epidemiological evidence, and possible biological mechanisms. Leuk Res 121: 106924. 10.1016/j.leukres.2022.106924

91. Marques-Mourlet C, Di Iorio R, Fairfield H, and Reagan MR (2023) Obesity and myeloma: Clinical and mechanistic contributions to disease progression. Front Endocrinol (Lausanne) 14: 1118691. 10.3389/fendo.2023.1118691

92. Martinez-Herrera M, Silvestre-Rangil J, and Silvestre FJ (2017) Association between obesity and periodontal disease. A systematic review of epidemiological studies and controlled clinical trials. Med Oral Patol Oral Cir Bucal 22(6): e708-e715. 10.4317/medoral.21786

93. Matos A, Marinho-Dias J, Ramalheira S, Oliveira MJ, Bicho M, and Ribeiro R (2016) Mechanisms underlying the association between obesity and Hodgkin lymphoma. Tumour Biol 37(10): 13005-13016. 10.1007/s13277-016-5198-4

94. McCreadie RG and Scottish Schizophrenia Lifestyle G (2003) Diet, smoking and cardiovascular risk in people with schizophrenia: descriptive study. Br J Psychiatry 183: 534-9. 10.1192/bjp.183.6.534

95. McElroy SL and Keck PE, Jr. (2012) Obesity in bipolar disorder: an overview. Curr Psychiatry Rep 14(6): 650-8. 10.1007/s11920-012-0313-8

96. Misicka E, Gunzler D, Albert J, and Briggs FBS (2023) Characterizing causal relationships of visceral fat and body shape on multiple sclerosis risk. Mult Scler Relat Disord 79: 104964. 10.1016/j.msard.2023.104964

97. Morimoto Y, Takahashi H, Fujii M, Miyoshi N, Uemura M, Matsuda C, Yamamoto H, Mizushima T, Mori M, and Doki Y (2019) Visceral obesity is a preoperative risk factor for postoperative ileus after surgery for colorectal cancer: Single-institution retrospective analysis. Ann Gastroenterol Surg 3(6): 657-666. 10.1002/ags3.12291

98. Murphy L, Schwartz TA, Helmick CG, Renner JB, Tudor G, Koch G, Dragomir A, Kalsbeek WD, Luta G, and Jordan JM (2008) Lifetime risk of symptomatic knee osteoarthritis. Arthritis Rheum 59(9): 1207-13. 10.1002/art.24021

99. Nam GE, Cho KH, Han K, Kim CM, Han B, Cho SJ, Jung SJ, Kwon Y, Kim YH, Kim DH, Kim SM, Choi YS, Roh YK, and Park YG (2019) Obesity, abdominal obesity and subsequent risk of kidney cancer: a cohort study of 23.3 million East Asians. Br J Cancer 121(3): 271-277. 10.1038/s41416-019-0500-z

100. Ng Yin Ling C, Lim SC, Jonas JB, and Sabanayagam C (2021) Obesity and risk of age-related eye diseases: a systematic review of prospective population-based studies. Int J Obes (Lond) 45(9): 1863-1885. 10.1038/s41366-021-00829-y

101. Ni Mhurchu C, Rodgers A, Pan WH, Gu DF, Woodward M, and Asia Pacific Cohort Studies C (2004) Body mass index and cardiovascular disease in the Asia-Pacific Region: an overview of 33 cohorts involving 310 000 participants. Int J Epidemiol 33(4): 751-8. 10.1093/ije/dyh163

102. Nitsche LJ, Mukherjee S, Cheruvu K, Krabak C, Rachala R, Ratnakaram K, Sharma P, Singh M, and Yendamuri S (2022) Exploring the Impact of the Obesity Paradox on Lung Cancer and Other Malignancies. Cancers (Basel) 14(6). 10.3390/cancers14061440

103. Nujic D, Music Milanovic S, Milas V, Miskulin I, Ivic V, and Milas J (2021) Association between child/adolescent overweight/obesity and conduct disorder: A systematic review and meta-analysis. Pediatr Obes 16(5): e12742. 10.1111/ijpo.12742

104. Oktarina C, Surya D, Sukma PMG, Manurung THP, and Widaty S (2021) Association between nutritional status and scabies infestation in a boarding school in Indonesia: a cross sectional study. Iranian Journal of Dermatology 24(4): 280-285. 10.22034/ijd.2020.241529.1179

105. Onstad MA, Schmandt RE, and Lu KH (2016) Addressing the Role of Obesity in Endometrial Cancer Risk, Prevention, and Treatment. J Clin Oncol 34(35): 4225-4230. 10.1200/JCO.2016.69.4638

106. Pan A, Sun Q, Czernichow S, Kivimaki M, Okereke OI, Lucas M, Manson JE, Ascherio A, and Hu FB (2012) Bidirectional association between depression and obesity in middle-aged and older women. Int J Obes (Lond) 36(4): 595-602. 10.1038/ijo.2011.111

107. Pandey S and Bhattacharya S (2010) Impact of obesity on gynecology. Womens Health (Lond) 6(1): 107-17. 10.2217/whe.09.77

108. Perez-Bonaventura I, Granero R, and Ezpeleta L (2015) The relationship between weight status and emotional and behavioral problems in Spanish preschool children. J Pediatr Psychol 40(4): 455-63. 10.1093/jpepsy/jsu107

109. Picon-Ruiz M, Morata-Tarifa C, Valle-Goffin JJ, Friedman ER, and Slingerland JM (2017) Obesity and adverse breast cancer risk and outcome: Mechanistic insights and strategies for intervention. CA Cancer J Clin 67(5): 378-397. 10.3322/caac.21405

110. Pipitone RM, Ciccioli C, Infantino G, La Mantia C, Parisi S, Tulone A, Pennisi G, Grimaudo S, and Petta S (2023) MAFLD: a multisystem disease. Ther Adv Endocrinol Metab 14: 20420188221145549. 10.1177/20420188221145549

111. Poudel D, George MD, and Baker JF (2020) The Impact of Obesity on Disease Activity and Treatment Response in Rheumatoid Arthritis. Curr Rheumatol Rep 22(9): 56. 10.1007/s11926-020-00933-4

112. Poynter JN, Richardson M, Blair CK, Roesler MA, Hirsch BA, Nguyen P, Cioc A, Warlick E, Cerhan JR, and Ross JA (2016) Obesity over the life course and risk of acute myeloid leukemia and myelodysplastic syndromes. Cancer Epidemiol 40: 134-40. 10.1016/j.canep.2015.12.005

113. Purdy JC and Shatzel JJ (2021) The hematologic consequences of obesity. Eur J Haematol 106(3): 306-319. 10.1111/ejh.13560

114. Quinones-Ossa GA, Lobo C, Garcia-Ballestas E, Florez WA, Moscote-Salazar LR, and Agrawal A (2021) Obesity and Stroke: Does the Paradox Apply for Stroke? Neurointervention 16(1): 9-19. 10.5469/neuroint.2020.00108

115. Radoi L, Paget-Bailly S, Cyr D, Papadopoulos A, Guida F, Tarnaud C, Menvielle G, Schmaus A, Cenee S, Carton M, Lapotre-Ledoux B, Delafosse P, Stucker I, and Luce D (2013) Body mass index, body mass change, and risk of oral cavity cancer: results of a large population-based case-control study, the ICARE study. Cancer Causes Control 24(7): 1437-48. 10.1007/s10552-013-0223-z

116. Ravanbakhsh S, Batech M, and Tejirian T (2015) Increasing Body Mass Index Is Inversely Related to Groin Hernias. Am Surg 81(10): 1043-6.

117. Reilly-Harrington NA, Feig EH, and Huffman JC (2018) Bipolar Disorder and Obesity: Contributing Factors, Impact on Clinical Course, and the Role of Bariatric Surgery. Curr Obes Rep 7(4): 294-300. 10.1007/s13679-018-0322-y

118. Renehan AG, Tyson M, Egger M, Heller RF, and Zwahlen M (2008) Body-mass index and incidence of cancer: a systematic review and meta-analysis of prospective observational studies. Lancet 371(9612): 569-78. 10.1016/S0140-6736(08)60269-X

119. Saha A, Kolonin MG, and DiGiovanni J (2023) Obesity and prostate cancer - microenvironmental roles of adipose tissue. Nat Rev Urol 20(10): 579-596. 10.1038/s41585-023-00764-9

120. Saitta C, Pollicino T, and Raimondo G (2019) Obesity and liver cancer. Ann Hepatol 18(6): 810-815. 10.1016/j.aohep.2019.07.004

121. Salas-Huetos A, Maghsoumi-Norouzabad L, James ER, Carrell DT, Aston KI, Jenkins TG, Becerra-Tomas N, Javid AZ, Abed R, Torres PJ, Luque EM, Ramirez ND, Martini AC, and Salas-Salvado J (2021) Male adiposity, sperm parameters and reproductive hormones: An updated systematic review and collaborative meta-analysis. Obes Rev 22(1): e13082. 10.1111/obr.13082

122. Sangani RG, Ghio AJ, Mujahid H, Patel Z, Catherman K, Wen S, and Parker JE (2021) Outcomes of Idiopathic Pulmonary Fibrosis Improve with Obesity: A Rural Appalachian Experience. South Med J 114(7): 424-431. 10.14423/SMJ.0000000000001275

123. Sansone RA and Sansone LA (2013) Obesity and substance misuse: is there a relationship? Innov Clin Neurosci 10(9-10): 30-5.

124. Sergentanis TN, Tsivgoulis G, Perlepe C, Ntanasis-Stathopoulos I, Tzanninis IG, Sergentanis IN, and Psaltopoulou T (2015) Obesity and Risk for Brain/CNS Tumors, Gliomas and Meningiomas: A Meta-Analysis. PLoS One 10(9): e0136974. 10.1371/journal.pone.0136974

125. Shaw E, Farris M, McNeil J, and Friedenreich C (2016) Obesity and Endometrial Cancer. Recent Results Cancer Res 208: 107-136. 10.1007/978-3-319-42542-9_7

126. Shimizu T, Kimura N, Mieno M, Hori D, Shiraishi M, Tashima Y, Yuri K, Itagaki R, Aizawa K, Kawahito K, and Yamaguchi A (2020) Effects of Obesity on Outcomes of Acute Type A Aortic Dissection Repair in Japan. Circ Rep 2(11): 639-647. 10.1253/circrep.CR-20-0098

127. Sohn W, Lee HW, Lee S, Lim JH, Lee MW, Park CH, and Yoon SK (2021) Obesity and the risk of primary liver cancer: A systematic review and meta-analysis. Clin Mol Hepatol 27(1): 157-174. 10.3350/cmh.2020.0176

128. Song RH, Wang B, Yao QM, Li Q, Jia X, and Zhang JA (2019) The Impact of Obesity on Thyroid Autoimmunity and Dysfunction: A Systematic Review and Meta-Analysis. Front Immunol 10: 2349. 10.3389/fimmu.2019.02349

129. Stefani C, Pecoraro L, Flodmark CE, Zaffanello M, Piacentini G, and Pietrobelli A (2023) Allergic Diseases and Childhood Obesity: A Detrimental Link? Biomedicines 11(7). 10.3390/biomedicines11072061

130. Strazzullo P, D'Elia L, Cairella G, Garbagnati F, Cappuccio FP, and Scalfi L (2010) Excess body weight and incidence of stroke: meta-analysis of prospective studies with 2 million participants. Stroke 41(5): e418-26. 10.1161/STROKEAHA.109.576967

131. Su PY, Hsu YC, Cheng YF, Kor CT, and Su WW (2019) Strong association between metabolically-abnormal obesity and gallstone disease in adults under 50 years. BMC Gastroenterol 19(1): 117. 10.1186/s12876-019-1032-y

132. Suren P, Gunnes N, Roth C, Bresnahan M, Hornig M, Hirtz D, Lie KK, Lipkin WI, Magnus P, Reichborn-Kjennerud T, Schjolberg S, Susser E, Oyen AS, Smith GD, and Stoltenberg C (2014) Parental obesity and risk of autism spectrum disorder. Pediatrics 133(5): e1128-38. 10.1542/peds.2013-3664

133. Tan W, Gao M, Liu N, Zhang G, Xu T, and Cui W (2015) Body Mass Index and Risk of Gallbladder Cancer: Systematic Review and Meta-Analysis of Observational Studies. Nutrients 7(10): 8321-34. 10.3390/nu7105387

134. Thandra KC, Barsouk A, Saginala K, Padala SA, Barsouk A, and Rawla P (2021) Epidemiology of Non-Hodgkin's Lymphoma. Med Sci (Basel) 9(1). 10.3390/medsci9010005

135. Tsigalou C, Vallianou N, and Dalamaga M (2020) Autoantibody Production in Obesity: Is There Evidence for a Link Between Obesity and Autoimmunity? Curr Obes Rep 9(3): 245-254. 10.1007/s13679-020-00397-8

136. Tzenios N, Tazanios ME, and Chahine M (2022) The impact of body mass index on prostate cancer: An updated systematic review and meta-analysis. Medicine (Baltimore) 101(45): e30191. 10.1097/MD.0000000000030191

137. Uhlenhopp DJ, Then EO, Sunkara T, and Gaduputi V (2020) Epidemiology of esophageal cancer: update in global trends, etiology and risk factors. Clin J Gastroenterol 13(6): 1010-1021. 10.1007/s12328-020-01237-x

138. Wertli MM, Held U, Campello M, and Schecter Weiner S (2016) Obesity is associated with more disability at presentation and after treatment in low back pain but not in neck pain: findings from the OIOC registry. BMC Musculoskelet Disord 17: 140. 10.1186/s12891-016-0992-0

139. Yang W, Zeng X, Petrick JL, Danford CJ, Florio AA, Lu B, Nan H, Ma J, Wang L, Zeng H, Sudenga SL, Campbell PT, Giovannucci E, McGlynn KA, and Zhang X (2022) Body mass index trajectories, weight gain and risks of liver and biliary tract cancers. JNCI Cancer Spectr 6(4). 10.1093/jncics/pkac056

140. Yang Y, Xian W, Wu D, Huo Z, Hong S, Li Y, and Xiao H (2022) The role of obesity, type 2 diabetes, and metabolic factors in gout: A Mendelian randomization study. Front Endocrinol (Lausanne) 13: 917056. 10.3389/fendo.2022.917056

141. Ye P, Xi Y, Huang Z, and Xu P (2020) Linking Obesity with Colorectal Cancer: Epidemiology and Mechanistic Insights. Cancers (Basel) 12(6). 10.3390/cancers12061408

142. Ye YM, Jin HJ, Hwang EK, Nam YH, Kim JH, Shin YS, and Park HS (2013) Co-existence of chronic urticaria and metabolic syndrome: clinical implications. Acta Derm Venereol 93(2): 156-60. 10.2340/00015555-1443

143. Yetman AT and McCrindle BW (2010) The prevalence and clinical impact of obesity in adults with Marfan syndrome. Can J Cardiol 26(4): 137-9. 10.1016/s0828-282x(10)70370-6

144. Zain MM and Norman RJ (2008) Impact of obesity on female fertility and fertility treatment. Womens Health (Lond) 4(2): 183-94. 10.2217/17455057.4.2.183

145. Zbiciak-Nylec M, Wcislo-Dziadecka D, Kasprzyk M, Kulig A, Laszczak J, Noworyta M, Adamus S, Brzezinska-Wcislo L, Rogala B, and Brzoza Z (2018) Overweight and obesity may play a role in the pathogenesis of chronic spontaneous urticaria. Clin Exp Dermatol 43(5): 525-528. 10.1111/ced.13368

146. Zendejas B, Hernandez-Irizarry R, Ramirez T, Lohse CM, Grossardt BR, and Farley DR (2014) Relationship between body mass index and the incidence of inguinal hernia repairs: a population-based study in Olmsted County, MN. Hernia 18(2): 283-8. 10.1007/s10029-013-1185-5

147. Zewari S, Hadi L, van den Elshout F, Dekhuijzen R, Heijdra Y, and Vos P (2018) Obesity in COPD: Comorbidities with Practical Consequences? COPD 15(5): 464-471. 10.1080/15412555.2018.1509951

148. Zhang A and Silverberg JI (2015) Association of atopic dermatitis with being overweight and obese: a systematic review and metaanalysis. J Am Acad Dermatol 72(4): 606-16 e4. 10.1016/j.jaad.2014.12.013

149. Zhang Z, Lai HJ, Roberg KA, Gangnon RE, Evans MD, Anderson EL, Pappas TE, Dasilva DF, Tisler CJ, Salazar LP, Gern JE, and Lemanske RF, Jr. (2010) Early childhood weight status in relation to asthma development in high-risk children. J Allergy Clin Immunol 126(6): 1157-62. 10.1016/j.jaci.2010.09.011

150. Zhou D, Wu J, and Luo G (2016) Body mass index and risk of non-melanoma skin cancer: cumulative evidence from prospective studies. Sci Rep 6: 37691. 10.1038/srep37691

151. Zucker I, Zloof Y, Bardugo A, Tsur AM, Lutski M, Cohen Y, Cukierman-Yaffe T, Minsky N, Derazne E, Tzur D, Melzer Cohen C, Pinhas-Hamiel O, Chodick G, Raz I, Afek A, Gerstein HC, Tirosh A, and Twig G (2022) Obesity in late adolescence and incident type 1 diabetes in young adulthood. Diabetologia 65(9): 1473-1482. 10.1007/s00125-022-05722-5
